# Determinants of tyrosinaemia during nitisinone therapy in alkaptonuria

**DOI:** 10.1038/s41598-022-20424-z

**Published:** 2022-09-27

**Authors:** L. R. Ranganath, A. M. Milan, A. T. Hughes, A. S. Davison, Khedr M, B. P. Norman, G. Bou-Gharios, J. A. Gallagher, R. Imrich, J. B. Arnoux, M. Rudebeck, B. Olsson

**Affiliations:** 1 Departments of Clinical Biochemistry and Metabolic Medicine, Royal Liverpool University Hospital, Liverpool University Hospitals NHS Foundation Trusts, Prescot Street, Liverpool, L7 8XP UK; 2 William Henry Duncan Building, University of Liverpool, Liverpool, UK; 3 Biomedical Research Centre, Slovak Academy of Sciences, Bratislava, Slovakia; 4 Hôpital Necker-Enfants Malades, Paris Cedex 15, France; 5 OnPoint Science AB, Stockholm, Sweden; 6 Garriguella AB, Ekerö, Sweden

**Keywords:** Biochemistry, Chemical biology, Physiology, Diseases, Molecular medicine, Pathogenesis

## Abstract

Nitisinone (NIT) produces inevitable but varying degree of tyrosinaemia. However, the understanding of the dynamic adaptive relationships within the tyrosine catabolic pathway has not been investigated fully. The objective of the study was to assess the contribution of protein intake, serum NIT (sNIT) and tyrosine pathway metabolites to nitisinone-induced tyrosinaemia in alkaptonuria (AKU). Samples of serum and 24-h urine collected during SONIA 2 (Suitability Of Nitisinone In Alkaptonuria 2) at months 3 (V2), 12 (V3), 24 (V4), 36 (V5) and 48 (V6) were included in these analyses. Homogentisic acid (HGA), tyrosine (TYR), phenylalanine (PHE), hydroxyphenylpyruvate (HPPA), hydroxyphenyllactate (HPLA) and sNIT were analysed at all time-points in serum and urine. Total body water (TBW) metabolites were derived using 60% body weight. 24-h urine and TBW metabolites were summed to obtain combined values. All statistical analyses were post-hoc. 307 serum and 24-h urine sampling points were analysed. Serum TYR from V2 to V6, ranging from 478 to 1983 µmol/L were stratified (number of sampling points in brackets) into groups < 701 (47), 701–900 (105), 901–1100 (96) and > 1100 (59) µmol/L. The majority of sampling points had values greater than 900 µmol/L. sPHE increased with increasing sTYR (*p* < 0.001). Tyrosine, HPPA and HPLA in serum and TBW all increased with rising sTYR (*p* < 0.001), while HPLA/TYR ratio decreased (*p* < 0.0001). During NIT therapy, adaptive response to minimise TYR formation was demonstrated. Decreased conversion of HPPA to HPLA, relative to TYR, seems to be most influential in determining the degree of tyrosinaemia.

## Introduction

In alkaptonuria (AKU) (OMIM#203500), a disorder of the tyrosine (TYR) pathway, the lack of homogentisate 1,2 dioxygenase (HGD) (EC.1.13.11.5) activity results in failure to convert homogentisic acid (HGA) to maleylacetoacetic acid, thus leading to accumulation of HGA and the damaging effects of AKU^1,2^. Nitisinone (NIT) is approved by the European Medicines Agency as the first disease-modifying therapy for AKU^3,4^. It decreases HGA^5^ by inhibiting 4-hydroxyphenylpyruvate dioxygenase (HPPD, EC:1.13.11.27) and consequently ameliorates AKU^6^. However, inhibition of HPPD also leads to accumulation of metabolites proximal to this inhibition^7^, including TYR leading to severe tyrosinaemia which can cause unwanted effects such as corneal keratopathy, and, as more recently reported, vitiligo and cataract formation^8–10^. Tyrosinaemia has also been potentially linked with the cognitive impairment observed in children treated with life-saving NIT in hereditary tyrosinaemia type 1 (HT-1) (OMIM#276700)^11^, however research is currently ongoing to determine the degree and cause of these findings. Values for circulating TYR are typically between 1500 and 2500 µmol/L in tyrosinaemias, especially due to nitisinone-mediated inhibition of HPPD^5,12^. Fasting TYR concentrations in normal adults have been determined to be between 30 and 86 µmol/L^13^.

In order to meet the requirements for sufficient intake of the limiting amino acids tryptophan, methionine, threonine and lysine, humans consume protein that results in surplus TYR and phenylalanine (PHE)^14^. The average protein intake in many Western countries is 150–200% of recommended values^15,16^. While TYR is consumed through the diet, it can also be generated within the body by conversion of the essential amino acid PHE to TYR through the action of phenylalanine hydroxylase (EC 1.14. 16.1) (Fig. S1). Excess PHE and TYR, like other amino acids, cannot be stored and are instead catabolised. Therefore, the excess consumption of PHE and TYR, while not a problem when the TYR pathway is normal, could contribute to the tyrosinaemia during NIT therapy, but a thorough analysis of such contribution is not available.

PHE, TYR, hydroxyphenylpyruvate (HPPA) and hydroxyphenyllactate (HPLA) are among the main tyrosine pathway metabolites proximal to HPPD inhibition that could be altered during NIT treatment^7^. In the previous paper^17^ the increase per metabolite was determined, however, the understanding of the dynamic adaptive relationships was not investigated fully. In the phase 3 Suitability of Nitisinone in Alkaptonuria 2 study (SONIA 2)^4^, a dose of NIT of 10 mg daily was administered by the oral route to all nitisinone-randomised patients. This is different from NIT therapy for HT-1, where the most common daily dose is between 1 mg/kg body weight, with dietetic restriction of TYR and PHE^18^. As far as we are aware a correlative analysis of the relationship between sNIT and tyrosinaemia including sTYR has not yet been carried out.

The complete TYR pathway is expressed mainly in the liver and to some extent in the kidney^19^. In untreated AKU, HGA is almost exclusively produced in the liver and kidney^19^. During NIT therapy, the inhibition of HPPD in the liver and kidney results in an overproduction of metabolites proximal to HGA in these organs. Once produced, HGA in untreated AKU, and HPPA, HPLA, and TYR during NIT treatment, are exported from these organs. The PHE/TYR metabolites produced in the liver reach the systemic circulation and whole-body water before being excreted in the urine. In the kidney, HGA in the untreated AKU state and HPPA and HPLA during NIT therapy are overproduced; it is assumed that these metabolites are excreted in the urine rather than being absorbed back into the body^20^.

The tyrosine pathway results in the disposal of excess PHE and TYR. Although HPPA is formed from TYR, it can also be reconverted back to TYR by the bidirectional nature of tyrosine aminotransferase (TAT) (Fig. S1); additionally, HPPA can be converted to HPLA. During NIT therapy the route of further metabolism of the accumulating HPPA could determine the magnitude of tyrosinaemia, but such an analysis has yet to be carried out. The aim of the present analyses is therefore to study the factors that influence tyrosinaemia during NIT therapy such as protein intake, NIT itself and metabolic pathway adaptations.

## Methods

### Study design and patients

SONIA 2 was a four-year, open-label, evaluator-blinded, multicentre, randomised, no-treatment controlled, parallel-group study as previously described^4^. The study design is summarised in Fig. S2. The study was performed at three investigational sites: Liverpool (UK), Paris (France) and Piešťany (Slovakia). Independent Ethics Committee at each centre approved the study. The aim was to recruit 140 patients aged 25 years or older, with a confirmed diagnosis of AKU and any clinical manifestation in addition to increased HGA; 70 randomised to nitisinone and 70 to a control (no-nitisinone) group. All patients provided written informed consent prior to inclusion. Normal renal function defined by eGFR was a required inclusion criterion.

Oral nitisinone (Orfadin®) 10 mg daily was administered in the treated group. The control group did not receive the study drug. There were no restrictions regarding concomitant medications. Patients in both groups could freely use analgesics, anti-inflammatory drugs and others as needed to treat symptoms of AKU. Details of randomisation are discussed in detail in a previous publication^4^.

### Procedures

A number of assessments and investigations including collection of medical history and physical examination, including those specific for AKU, a wide range of clinical outcome measures of safety and efficacy were carried out and shown elsewhere^4^. Relevant to the analyses presented here; patients visited study sites at baseline (Visit 1; V1), 3 months (V2), and then annually up to month 48 (V3-V6); a close-out phone call took place at month 49. Only data from visits V2 to V6 in the nitisinone-receiving group will be discussed in the rest of the manuscript; V1 data are not used in the present study since no TYR (and metabolite) data were collected during NIT treatment at that visit. There was no active management of the diet during SONIA 2 study. Patients were given an information sheet to decrease protein intake to minimise tyrosinaemia during NIT therapy. The need to limit dietary protein intake was reinforced at each visit at all study sites.

At each visit, 24-h urine was collected for the measurement of urea, creatinine and phenylalanine/tyrosine pathway metabolites, into 2.5 L bottles containing 30 mL of 5 N H_2_SO_4_ and stored away from direct sunlight. The weight of the collected urine was recorded and used as the volume in the calculations of amount of urea excreted assuming a density of 1 g/mL. An aliquot of the collected urine was frozen and kept at − 20 °C until analysis.

### Chemical analyses

Measurement of NIT, HGA, TYR, PHE, HPPA, and HPLA, in serum (indicated as sNIT, sHGA, sTYR, sPHE, sHPPA, and sHPLA) and 24-h urine (indicated as uHGA_24_, uTYR_24_, uPHE_24_, uHPPA_24_, and uHPLA_24_) were carried out on all samples collected at the described study visits. Blood samples were collected in plain serum tubes (Sarstedt, Germany). An aliquot of serum was immediately acidified using perchloric acid (10% v/v 5.8 M)^21^, to stabilise the HGA, and kept frozen at − 80 °C until analysis. Samples from Paris and Piešťany were transported frozen by courier to Liverpool and all biochemical analyses were performed in the Department of Clinical Biochemistry, Liverpool Clinical Laboratories, Liverpool University Hospital NHS Foundation Trust.

The concentrations of sNIT, sHGA, sTYR, sPHE, sHPPA, and sHPLA as well as uHGA_24_, uTYR_24_, uPHE_24_, uHPPA_24_, and uHPLA_24_ were measured by liquid chromatography tandem mass spectrometry using previously published methods^7,17^. All analyses were performed on an Agilent 6490 Triple Quadrupole mass spectrometer with Jet-Stream electrospray ionisation coupled with an Agilent 1290 Infinity II Ultra-High Performance Liquid Chromatography system. Briefly, this method incorporates reverse-phase chromatographic separation on an Atlantis dC18 column (100 mm × 3.0 mm, 3 µm, Waters); initial chromatographic conditions of 80:20 water:methanol with 0.1% formic acid (v/v) increased linearly to 10:90 over 5 min. Matrix-matched calibration standards and quality controls were used with appropriate isotopic-labelled internal standards with quantification in multiple reaction mode (NIT, PHE and TYR in positive ionisation and HPPA, HPLA and HGA in negative ionisation). Sample preparation was by dilution in a combined internal standard solution containing ^13^C_6_-nitisinone, ^13^C_6_-HGA, d_4_-TYR and d_5_-PHE in 0.1% formic acid (v/v) in deionised water. No internal standard was available for HPPA and HPLA at time of analysis and ^13^C_6_-HGA was therefore validated for use as the internal standard.

Urine urea and creatinine were photometrically assayed in the 24-h urine collection on a Roche Cobas 701 using an automated assay (hydrolysis with urease and subsequent oxidation of NADH). Urine urea was used to objectively estimate dietary protein intake in keeping with other studies^22,23^. Urine creatinine was measured using a validated Jaffe reaction.

#### Total body water (TBW) content of HGA, TYR, PHE, HPPA and HPLA (tbwHGA, tbwTYR, tbwPHE, tbwHPPA, tbwHPLA)

Since PHE and TYR and their metabolites are small molecules that are distributed in TBW^24–26^, the concentration (µmol/L) of circulating metabolites was multiplied by TBW in litres (calculated as 0.6 times body weight in kilograms) to derive the amount of metabolites in TBW (µmol)^24–26^.

#### Total urinary metabolites

 TYR and PHE and their metabolites were quantitated and multiplied by the 24-h urine volumes to yield daily metabolite excretion (µmol/day) before and during treatment with nitisinone.

#### Combined metabolites of HGA, TYR, PHE, HPPA and HPLA (cHGA_24_, cTYR_24_, cPHE_24_, cHPPA_24_, and cHPLA_24_)

The metabolites in the TBW and 24-h urine were summed to obtain the total (called combined) metabolites generated daily in the liver and kidney.

### Statistical analysis

All statistical analyses were post-hoc. Continuous variables are presented using mean and standard deviation (SD). Analyses were performed using Graphpad InStat 3 software (version number 3.1); *p* values < 0.05 were considered statistically significant. In order to understand the characteristics of sTYR better, all sampling points in all nitisinone-treated patients were analysed with regard to sTYR relationships with other data in terms of differences in thresholds of sTYR both by ANOVA (Tukey–Kramer for multiple comparisons) and simple linear regression.

### Role of the funding source

This study was funded by a grant from the European Union Framework Programme 7 (DevelopAKUre, project number: 304985). The funder of the study had no role in study design, data collection, data analysis, data interpretation, or writing of the report. The corresponding author had full access to all the data in the study and had final responsibility for the decision to submit for publication.

## Results

### Demographics

In SONIA 2, 69 patients were randomly assigned to receive nitisinone 10 mg daily and 69 to no treatment. Of these, 108 patients completed the study. The main reason for discontinuation in the control group was withdrawal of consent (n = 10), with adverse event corneal keratopathy, (n = 9) being the most common reason in the nitisinone group^4^. Demographic data and baseline characteristics for the 69 NIT-treated patients are shown in Table S1. There were a number of concomitant medications used by patients during the study period and shown as a supplementary file. Hypothyroid patients were on replacement and assumed to be euthyroid since thyroid function was not tested during the SONIA 2 study.

### Distribution of sTYR in patients receiving nitisinone

**Figure 1:**
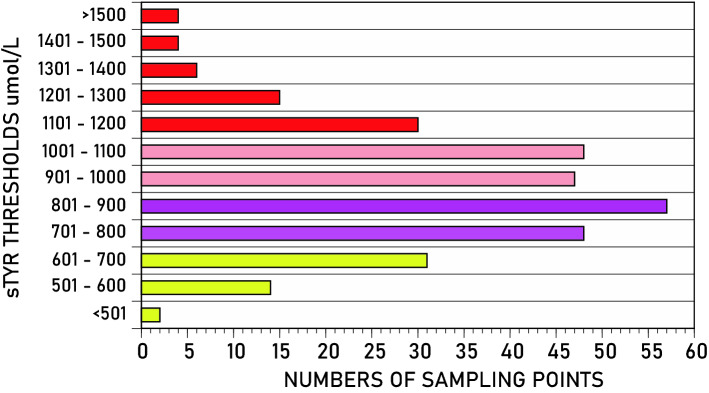
Distribution of sTYR concentrations between V2 and V6 in NIT-treated patients. The colour coding indicates numbers of sampling points below sTYR < 701 (yellow), 701–900 (violet), 901–1100 (pink) and > 1100 (red) µmol/L.

Out of a possible 345 serum samples between V2 and V6 in patients compliant with NIT therapy, 28 samples were not collected due to early withdrawal and a further 10 samples were excluded from the analysis due to poor compliance (no or very low NIT in serum) The remaining 307 samples were used in our analysis. Figure 1 shows the distribution of sTYR across these samples. The highest and lowest sTYR at any time were 1983 and 478 µmol/L respectively, with the mean (SD) of 924 (222) µmol/L; there were 152 samples with sTYR concentrations less than 900 µmol/L and 155 samples with more than 900 µmol/L. The data were grouped into four sTYR intervals. There were 47, 105, 96 and 59 samples in the < 701, 701–900, 901–1100 and > 1100 µmol/L groups respectively. All patients in SONIA 2 had normal renal function. There were 2 sample points less than 501 µmol/L and these were merged with the < 701 group.

### Metabolites changes across sTYR groups

*HGA measurements:* cHGA_24_, sHGA, tbwHGA, uHGA_24_ were all similar in all four sTYR groups (Tables 1, 2, Fig. S3).

**Table 1:** Measured metabolic data stratified according to sTYR groups in NIT-treated AKU patients. Demographic and measured data are shown according to sTYR intervals of < 701, 701–900, 901–1100 and > 1100 µmol/L.

	sTYR intervals			
	< 701 µmol/L (n = 46)	701–900 µmol/L (n = 103)	901–1100 µmol/L (n = 95)	> 1100 µmol/L (n = 59)
Age years	52.2 (10.7)	49.3 (11.8)	51.3 (10)	51.3 (11.5)
Weight kg	78 (13.8)	77.7 (13.6)	79 (16.2)	75.9 (18.3)
uUREA_24_ mmol/day	278 (147)	276 (136)	314 (144)	307 (150)
uUREA mmol/Kg	3.64 (1.93)	3.61 (1.92)	4.04 (1.74)	4.04 (1.7)
uCREAT_24_ mmol/day	11.4 (7.1)	10.6 (5.7)	10.9 (6.1)	10.3 (9)
sHGA µmol/L	0.9 (1.73)	0.91 (1.28)	0.74 (0.93)	0.87 (1.55)
sTYR µmol/L****	624 (65)	805 (55)	1001 (58)	1247 (175)
sPHE µmol/L****	58.6 (12.6)	61.7 (10.5)	62.7 (11.4)	69.9 (20.6)
sHPPA µmol/L L****	33.8 (6.9)	36.6 (11.2)	40.8 (26.3)	47.6 (14.8)
sHPLA µmol/L****	68.6 (19.8)	82.4 (28.7)	93.6 (21.7)	121.3 (35.4)
sNIT µmol/L	4.79 (2.25)	4.587 (2.68)	4.84 (2.56)	5.73 (3.09)
uHGA_24_ µmol/day	194 (408)	270 (540)	219 (368)	169 (183)
uTYR_24_ µmol/day	1138 (622)	1309 (740)	1354 (777)	1516 (952)
uPHE_24_ µmol/day	65 (37)	63 (34)	59 (43)	64 (42)
uHPPA_24_ µmol/day	15,633 (8696)	15,545 (7323)	17,271 (8845)	18,056 (7945)
uHPLA_24_ µmol/day	12,647 (5974)	13,277 (5112)	15,526 (5880)	16,453 (7016)
sHGA/sTYR*	0.0013 (0.0023)	0.0011 (0.0016)	0.0007 (0.0009)	0.0007 (0.001)
sTYR/sPHE****	11.18 (2.82)	13.43 (2.5)	16.47 (3.07)	18.9 (4.46)
sHPPA/sTYR****	0.055 (0.012)	0.046 (0.014)	0.041 (0.028)	0.038 (0.011)
sHPPA/sHPLA**	0.52 (0.15)	0.47 (0.14)	0.45 (0.28)	0.4 (0.1)
sHPLA/sTYR**	0.111 (0.03)	0.102 (0.03)	0.094 (0.02)	0.097 (0.02)
uHGA_24_/uTYR_24_	0.165 (0.292)	0.212 (0.394)	0.154 (0.198)	0.117 (0.126)
uTYR_24_/uPHE_24_	18.21 (4.77)	21.98 (5.31)	24.07 (5.2)	25.64 (5.6)
uHPPA_24_/uTYR_24_	16.33 (10.4)	15.33 (10.2)	14.81 (6.74)	15.29 (9.63)
uHPPA_24_/uHPLA_24_	1.25 (0.35)	1.19 (0.37)	1.12 (0.33)	1.12 (0.33)
uHPLA_24_/uTYR_24_***	14.65 (15.4)	14.22 (11.1)	14.03 (6.8)	14.4 (9.2)

Variation among column means is significantly greater than expected by chance with *p* < : * < 0.05; ** < 0.01; *** < 0.001; **** < 0.0001; within sTYR group comparisons are shown in figures (main and supplementary).

S, serum; uX_24_, 24-h urine; HGA, homogentisic acid; TYR, tyrosine; PHE, phenylalanine; HPPA, 4-hydroxyphenylpyruvate; HPLA, 4-hydroxyphenyllactate; NIT, nitisinone; CREAT, creatinine.

**Table 2:** Derived metabolic data stratified according to sTYR groups in NIT-treated AKU patients. The total body water (TBW) and combined (total body water and 24-h urine) derived data are shown according to sTYR intervals of < 701, 701–900, 901–1100 and > 1100 µmol/L.

	sTYR intervals			
	< 701 umol/L (n = 46)	701–900 umol/L (n = 103)	901–1100 umol/L(n = 95)	> 1100 umol/L (n = 59)
tbwHGA µmol	44.3 (82.7)	43.9 (61.6)	35.4 (43.5)	41.8 (74.7)
tbwTYR µmol****	29,071 (5190)	37,502 (6839)	47,374 (9953)	56,528 (14,052)
tbwPHE µmol	2785 (875)	2913 (816)	3002 (909)	3204 (1249)
tbwHPPA µmol****	1598 (476)	1701 (534)	1927 (1180)	2187 (968)
tbwHPLA µmol****	3187 (944)	3808 (1263)	4463 (1568)	5532 (2055)
cHGA µmol	238 (469)	311 (573)	252 (397)	211 (229)
cTYR µmol****	30,062 (5486)	38,799 (7098)	48,714 (10,178)	58,044 (14,439)*
cPHE µmol	2803 (854)	2987 (837)	3069 (928)	3316 (1290)
cHPPA µmol	17,221 (8820)	17,096 (7560)	19,018 (9239)	20,242 (8294)
cHPLA µmol****	15,798 (6519)	16,957 (5681)	19,828 (6677)	21,985 (8036)*
tbwHGA/tbwTYR*	0.0014 (0.003)	0.0011 (0.0016)	0.0007 (0.0009)	0.0007 (0.001)
tbwTYR/tbwPHE****	11.18 (2.8)	13.39 (2.5)	16.47 (3.1)	18.9 (4.5)
tbwHPPA/tbwTYR****	0.055 (0.013)	0.046 (0.014)	0.041 (0.028)	0.038 (0.011)
tbwHPPA/tbwHPLA*	0.53 (0.15)	0.47 (0.14)	0.45 (0.28)	0.40 (0.1)
tbwHPLA/tbwTYR**	0.111 (0.03)	0.103 (0.03)	0.094 (0.02)	0.10 (0.02)
cHGA/cTYR	0.0074 (0.014)	0.0079 (0.015)	0.0052 (0.008)	0.0036 (0.004)
cTYR/cPHE****	11.43 (2.72)	13.56 (2.42)	16.57 (3.07)	18.9 (4.47)*
cHPPA/cTYR****	0.572 (0.26)	0.446 (0.2)	0.397 (0.18)	0.357 (0.14)*
cHPPA/cHPLA*	1.097 (0.3)	1.016 (0.31)	0.957 (0.3)	0.929 (0.25)*
cHPLA/cTYR****	0.532 (0.21)	0.443 (0.15)	0.412 (0.12)	0.382 (0.11)*

Variation among column means is significantly greater than expected by chance with *p* < : * < 0.05; ** < 0.01; *** < 0.001; **** < 0.0001; within sTYR group comparisons are shown in figures (main and supplementary.

TBW, total body water; c represents combined TBW plus 24-h urine values; HGA, homogentisic acid; TYR, tyrosine; PHE, phenylalanine; HPPA, 4-hydroxyphenylpyruvate; HPLA, 4-hydroxyphenyllactate.

*TYR measurements*: cTYR_24_, sTYR, and tbwTYR, but not uTYR_24_, significantly higher than in all sTYR groups. All sTYR groups were significantly different with respect to cTYR_24_, and tbwTYR when compared with each other (*p* < 0.0001 for all) (Tables 1, 2, Fig. S4).

**Figure 2:**
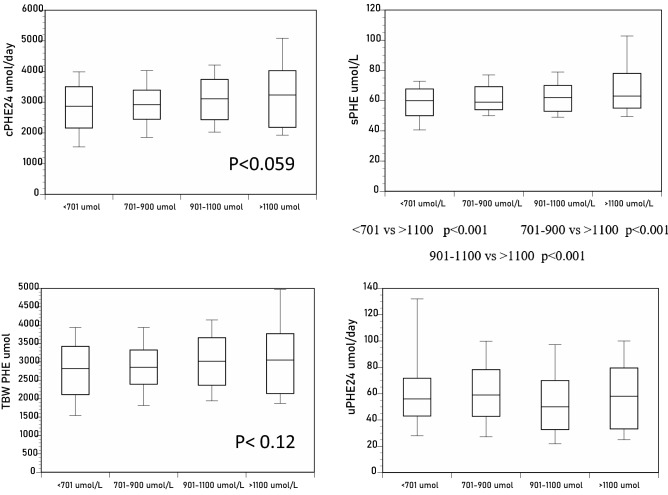
Differences in sPHE, uPHE_24_, tbwPHE, and cPHE_24_ across the sTYR groups of < 701, 701–900, 901–1100 and > 1100 µmol/L (*p* values indicated for within-group comparison where statistical significance was achieved).

*PHE measurements*: The group of sTYR greater than 1100 µmol/L showed higher sPHE compared to the other three groups (*p* < 0.001), with a similar trend for cPHE_24_ (*p* < 0.059) and tbwPHE (*p* < 0.12), but not uPHE_24_ (Tables 1, 2, Fig. 2).

*HPPA measurements*: The group of sTYR greater than 1100 µmol/L showed higher sHPPA than the two lowest groups < 701 and 701–900 µmol/L (*p* < 0.001), with tbwHPPA (*p* < 0.12) and cHPPA_24_ (*p* < 0.059) showing a similar trend (Tables 1, 2, Fig. S5).

*HPLA measurements*: All sTYR groups were significantly different from each other with regard to sHPLA, uHPLA_24_, tbwHPLA, and cHPLA_24_, with the 1100 µmol/L having the highest values (*p* < 0.001) (Tables 1, 2, Fig. S6). Groups < 701 and 701–900 showed both lower uHPLA_24_ and cHPLA_24_ compared to groups 901–1100 and > 1100.

### Metabolite ratios changes across sTYR groups

*HGA/TYR ratio*: None of the sHGA/sTYR, cHGA_24_/cTYR_24_ and uHGA_24_/uTYR_24_ ratios differed significantly across sTYR groups (Tables 1, 2, Fig. S7).

*TYR/PHE ratio*: cTYR_24_/cPHE_24_, sTYR/sPHE, tbwTYR/tbwPHE and uTYR_24_/uPHE_24_ ratios increased with increasing sTYR (Tables 1, 2, Fig. S8).

**Figure 3:**
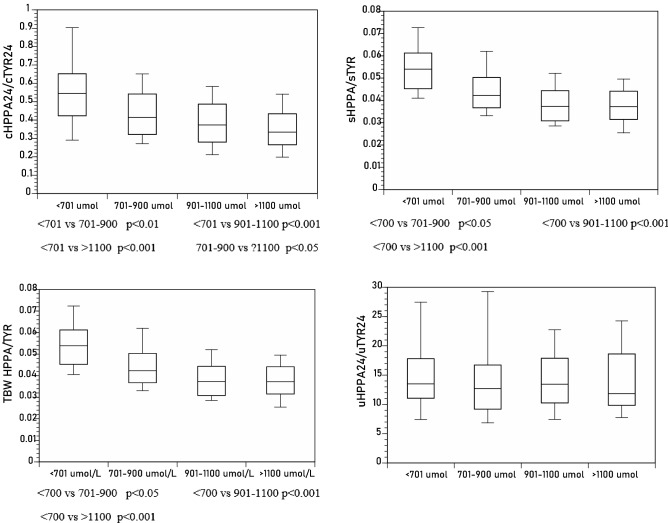
Differences in sHPPA/sTYR, uHPPA_24_/uTYR_24_, tbwHPPA/tbwTYR, and cHPPA_24_/cTYR_24_ across the sTYR groups of < 701, 701–900, 901–1100 and > 1100 µmol/L (*p* values indicated for within-group comparison where statistical significance was achieved).

*HPPA/TYR ratio*: cHPPA_24_/cTYR_24_, sHPPA/sTYR, and tbwHPPA/tbwTYR, but not the uHPPA_24_/uTYR_24_ ratio, decreased significantly with increasing sTYR (Tables 1, 2, Fig. 3).

**Figure 4:**
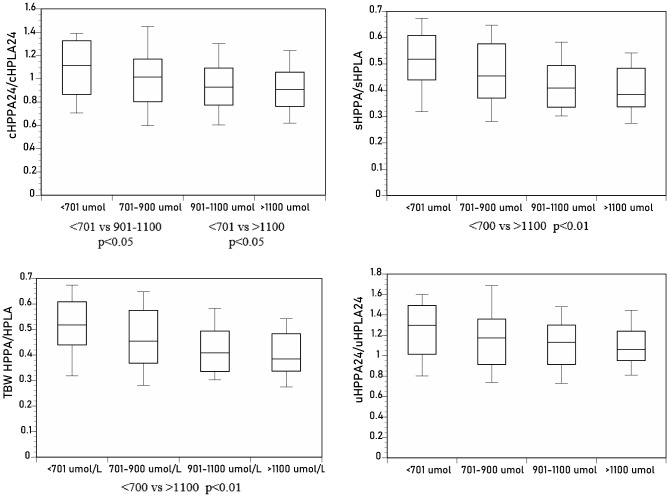
Differences in sHPPA/sHPLA, uHPPA_24_/uHPLA_24_, tbwHPPA/tbwHPLA, and cHPPA_24_/cHPLA_24_ across the sTYR groups of < 701, 701–900, 901–1100 and > 1100 µmol/L (*p* values indicated for within-group comparison where statistical significance was achieved).

*HPPA/HPLA ratio*: cHPPA_24_/cHPLA_24_, sHPPA/sHPLA, and tbwHPPA/tbwHPLA, but not the uHPPA_24_/uHPLA_24_ ratio, decreased significantly with increasing sTYR (Tables 1, 2, Fig. 4).

**Figure 5:**
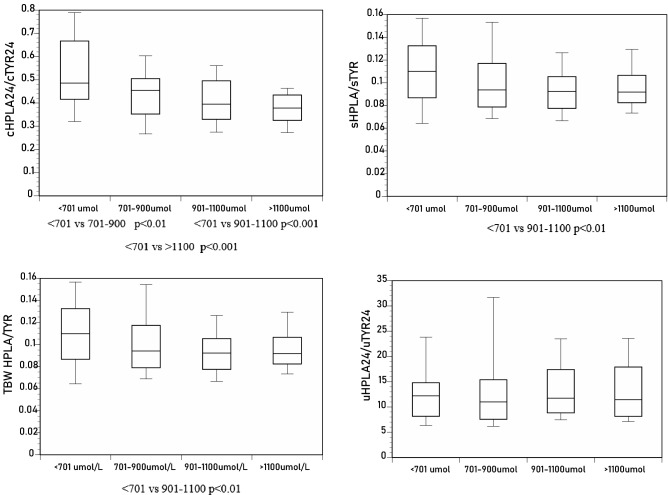
Differences in sHPLA/sTYR, uHPLA_24_/uTYR_24_, tbwHPLA/tbwTYR, and cHPLA_24_/cTYR_24_ across the sTYR groups of < 701, 701–900, 901–1100 and > 1100 µmol/L (*p* values indicated for within-group comparison where statistical significance was achieved).

*HPLA/TYR ratio*: cHPLA_24_/cTYR_24_, sHPLA/sTYR, and tbwHPLA/tbwTYR, but not uHPLA_24_/uTYR_24_ ratios, decreased significantly with increasing sTYR (Tables 1, 2, Fig. 5).

There were no differences in the *uUREA*_*24*_* mmol/day or uUREA*_*24*_* mmol/kg body weight* (Tables 1, Fig. S9) between any of the four sTYR groups.

### Regression analysis relationships between data during nitisinone-induced tyrosinaemia sTYR regression relationships

sTYR showed a weak positive relationship with uUREA_24_ (R 0.12). sTYR showed a significant positive relationship between sPHE (R 0.31), sHPPA (R 0.28) and sHPLA (R 0.59), strongest with sHPLA. There was a weak positive relationship between sTYR and sNIT (R 0.21) (Tables S2, S3).

sTYR showed a modest positive relationship with uHPLA_24_ (R 0.23) but not any other urine metabolites (Tables S2, S3).

sTYR showed the strongest but still modest relationship with cHPLA_24_ (R 0.31) with weaker relationships with other combined metabolites.

sTYR showed the strongest positive relationship with tbwTYR (R 0.71), and more modest positive relationships with tbwPHE (R 0.14), tbwHPPA (R 0.22), and tbwHPLA (R 0.47) (Tables S2, S3).

### uUREA_24_ linear regression relationships

uUREA_24_ showed a weak positive relationship with circulating metabolites (sHGA: R 0.15; sTYR: R 0.12; sPHE: R 0.16). uUREA_24_ showed a modest negative relationship with sNIT (R − 0.29) (Tables S2, S3).

Most urine metabolites were strongly positively related to uUREA_24_ (uTYR_24_: R 0.59; uPHE_24_: R 0.54; uHPPA_24_: R 0.8; uHPLA_24_: R 0.75) (Tables S2, S3).

uUREA_24_ showed strong relationships with cHPPA_24_ (R 0.79) and cHPLA_24_ (R 0.72). uUREA_24_ showed positive relationships with tbwHGA (R 0.16), tbwTYR (R 0.28), tbwPHE (R 0.26), tbwHPPA (R 0.14) and tbwHPLA (R 0.23) (Tables S2, S3).

### sNIT regression relationships

In relation to sNIT, there was a modest positive relationship with sTYR (R 0.21), but a stronger one with sHPLA (R 0.51). Age was positively associated with sNIT (R 0.34). sNIT was inversely related to body weight (R − 0.42) (Tables S2, S3).

## Discussion

Results from 307 samples were stratified into groups (sTYR intervals) (Fig. 1). The sTYR distribution in SONIA 2 patients on NIT in the < 501, 501–700, 701–900, 901–1100, and > 1100 µmol/L intervals was 0.65, 14.70, 34.31, 31.05 and 19.28% respectively, with 50.33% of the values greater than 900 µmol/L. The value of < 501 µmol/L refers to the acceptable value which requires no further action in the United Kingdom National Alkaptonuria Centre (Table S5). Surprisingly, a majority of subjects did not develop adverse consequences despite highly elevated levels. In fact, the highest sTYR of 1983 µmol/L was seen in an asymptomatic patient. We have, however, previously found a tendency towards more keratopathies with increasing sTYR levels^4^. Management of NIT-induced tyrosinaemia is challenging in adults due to the severely restricted diet needed affecting patient’s quality of life and optimal sTYR not being achievable without PHE/TYR-free amino acid supplements. In the United Kingdom National Alkaptonuria Centre sTYR intervals are used pragmatically. Protein intake is reduced to 0.9 and 0.8 g/kg body weight for sTYR values between 501–700, and 701–900 µmol/L respectively, whereas in those with values greater than 900 µmol/L, additional PHE/TYR free amino acid supplements are also employed (Table S4). These intervals are used because sTYR is associated with the increase in ocular TYR, and exhibits a known relationship to it^27–29^.

The fact that the HGD gene and the full tyrosine pathway are almost exclusively expressed in the liver and kidney formed the basis for studying metabolites as serum concentrations, and amounts in total body water and 24-h urine, and the latter two also combined, in the current analyses. The combined metabolites (cHGA_24_, cHPPA_24_, cHPLA_24_, cPHE_24_ and cTYR_24_), derived by summing the whole-body water and 24-h urine metabolites, gives the total amount of metabolites produced daily and is influenced by the HGD and HPPD insufficiencies. The ease of renal elimination of metabolites, the hepatic overproduction, and degree of dilution in TBW determines their circulating concentrations.

HGA, HPPA, HPLA and TYR values are not increased without NIT, and sNIT was used as a measure of HPPD inhibition. Finally, the metabolites themselves could influence each other further determining the circulating concentration of these metabolites including sTYR.

Dietary PHE and TYR intake also affect metabolite accumulation in NIT-induced HPPD inhibition. Our results showing that the uUREA_24_ and uUREA_24_/kg body weight were similar across the tyrosine threshold groups, are noteworthy and suggest that the differences described were not mostly due to differences in dietary PHE and TYR intakes. This should not be surprising since even with formal dietetic input, achieving acceptably low sTYR is challenging and often only achieved by using PHE/TYR-free amino acid supplements; this is in keeping with individual patient metabolic factors playing a key determinant role. We have also previously shown that most sTYR increases during NIT treatment occurred at very low doses and then plateau despite further increases in dose^30,31^. It is likely that there is a threshold for dietary protein intake which, when exceeded, does not further worsen tyrosinaemia on its own. When humans are in a stable steady state, intake is balanced by output and in the case of PHE and TYR, intake of these dietary amino acids daily is matched by either complete metabolism in health or an equilibrium for elimination of intermediary metabolites reached during NIT therapy. Since the usual proportion of PHE and TYR in the human diet is 60:40^32^, PHE contributes significantly to the flux down the pathway in steady state, and adaptation of PHE catabolism during tyrosinaemia could be quantitatively significant.

**Figure 6:**
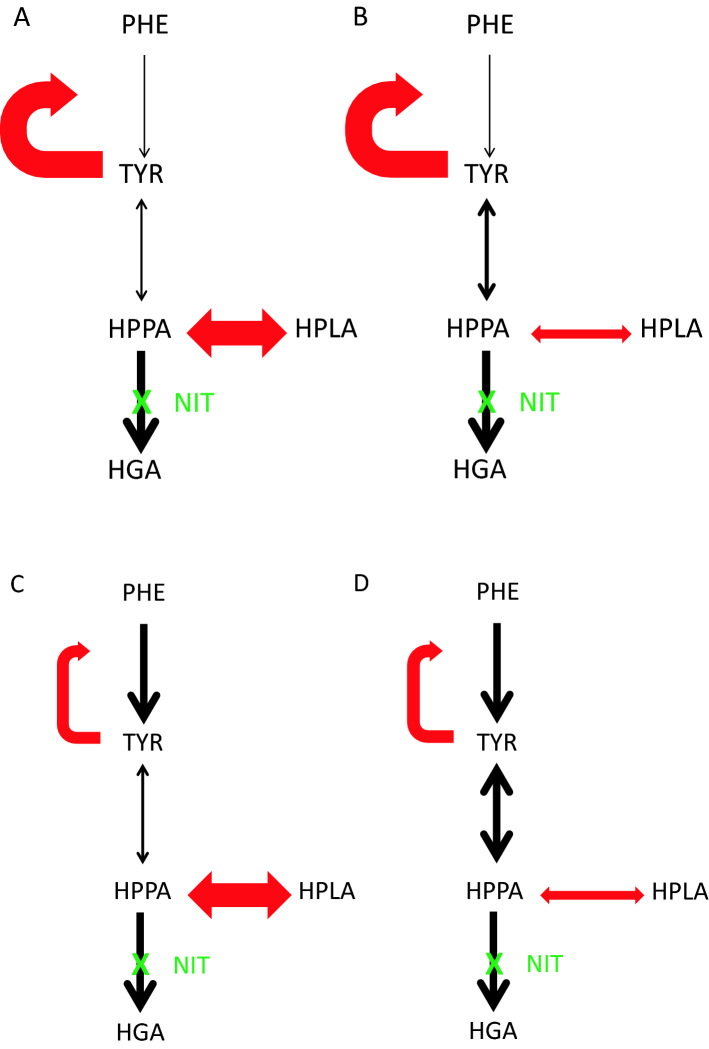
(**A**, **B**) A cartoon representation of changing relationships between PHE, TYR, HPPA and HPLA during nitisinone therapy. (**A**) Highlights the most adapted state showing lesser conversion of PHE to TYR (solid curved red arrow), and similarly higher conversion of HPPA to HPLA (a straight solid red arrow) as well as lower conversion between HPPA and TYR (a faintest straight black arrow). (**B**) Highlights an intermediate adapted state showing lesser conversion of PHE to TYR (solid curved red arrow), and lower conversion of HPPA to HPLA (a straight fainter red arrow) as well as an intermediate conversion between HPPA and TYR (the fainter straight black arrow). (**C**) Highlights an intermediate adapted state showing greater conversion of PHE to TYR (fainter curved red arrow), a higher conversion of HPPA to HPLA (a straight solid red arrow) as well as an intermediate conversion between HPPA and TYR (the fainter straight black arrow). (**D**) Highlights the least adapted state showing high conversion of PHE to TYR (fainter curved red arrow), a low conversion of HPPA to HPLA (a straight fainter red arrow) as well as lower conversion between HPPA and TYR (a solid straight black arrow).

sPHE was highest in the > 1100 µmol/L sTYR group suggesting high sTYR increases sPHE possibly by inhibiting the phenylalanine hydroxylase; this is consistent with pathway adaptation to minimise the formation of TYR from PHE, a beneficial change proposed by us recently^25^. sHPPA and sHPLA were highest in the sTYR > 1100 µmol/L group, especially the sHPLA, and is consistent with increased HPPA flux through the HPLA pathway also as suggested by us recently^27^. Urine 24-h metabolites were similar across the sTYR threshold groups except for uHPLA_24_ where the two highest groups (901–1100 and > 1100 µmol/L) were statistically higher than the two lowest groups (< 701 and 701–900 µmol/L), consistent with the importance of the HPLA generation. Combined metabolites represent the daily generation of metabolites in the liver and kidney, and shows convincingly significantly increased formation of TYR and HPLA from HPPA upon HPPD inhibition; the sHPPA/sTYR and cHPPA_24_/cTYR_24_ as well as sHPPA/sHPLA and cHPPA_24_/cHPLA_24_ decreased with increasing sTYR indicating that TYR and HPLA formation was greater than HPPA during NIT treatment. Although increased HPPA during NIT treatment resulted in increases in both TYR and HPLA, it is the relative change that is important. The % changes across the four sTYR groups from < 701 to > 1100 µmol/L in HPPA/TYR for serum, TBW, 24-h urine and all combined were 30.9, 30.9, 9.3 and 37.6% respectively, consistent with increasing TYR generation from HPPA. Likewise, the % changes across the four sTYR groups from < 701 to > 1100 µmol/L in HPPA/HPLA for serum, TBW, 24-h urine and all combined were 12.6, 24.5, 10.4 and 15.3% respectively, lower than for HPPA/TYR. These changes support the idea of preferential conversion of HPPA to TYR rather than to HPLA with increasing sTYR, and conversely the idea of relative deficiency of HPLA generation from HPPA during NIT therapy resulting in more severe tyrosinaemia. A direct comparison of HPLA and TYR more importantly shows a decrease in sHPLA/sTYR and cHPLA_24_/cTYR_24_ ratios with increasing tyrosinaemia and suggests greater conversion of HPPA to TYR compared to HPLA. Again, consistent with a relative deficiency in HPLA formation with increasing TYR (Fig. 6A–D).

The conversion of HPPA to HPLA is driven by HPPA accumulation due to HPPD inhibition whereas the increase in PHE could be driven by increase in TYR inhibiting phenylalanine hydroxylase. In HT-3, an inherited deficiency of HPPD, both of these factors may also play a part in mitigating increase in sTYR. In HT-2 on the other hand, the formation of HPLA, catalysed by an oxidoreductase called 4-hydroxyphenyllactate:NAD^+^ oxidoreductase, also called HPPR^33,34^ is lacking and leaves only increase in PHE driven by the increase in TYR to minimise tyrosinaemia, the reason why HT-2 patients develop more severe tyrosinaemia than HT-3 patients^35^.

We have described two metabolic adaptations to nitisinone-induced tyrosinaemia namely increase in PHE and an increase in HPLA. Four possible combinations of these two results could influence the degree of tyrosinaemia: (1) increase in PHE with increase in HPLA (full adaptation), (2) increase in PHE with no increase in HPLA (partial adaptation), (3) no increase in PHE with increase in HPLA (partial adaptation) and finally (4) no increase in PHE with no increase in HPLA (no adaptation) (Fig. 6A–D). The adaptations are likely to be a continuum even though we have used stratified sTYR values in the present analysis. Further analysis is needed to see if a critical value for a metabolite or metabolite ratio could predict tyrosinaemia-related effects such as keratopathy so that such patients may be targeted for more active TYR management.

It is undeniable that it is the nitisinone-induced HPPD inhibition compounded by excess daily consumption of PHE/TYR that is responsible for the tyrosinaemia supported by relatively weaker regression coefficient values between sTYR and sNIT (R 0.21) and sTYR and uUREA_24_ (R 0.12); similar linear regression analysis between sHPLA and sTYR was much stronger (R 0.59) (Table S2). This is supportive of our suggestion that HPLA generation may be more important than dietary protein intake or degree of HPPD inhibition in generating tyrosinaemia.

sNIT was positively related to age (R 0.34) and inversely related to body weight (R − 0.42) which may be expected as the same dose (10 mg) was given to all patients, irrespective of body weight. Nitisinone dosing in children with HT-1 is based on body weight with the most common dose being 1 mg/kg body weight. In the current dataset there was a modest positive relationship between sNIT and sHPPA (R 0.18), and stronger positive relationship between sNIT and sHPLA (R 0.51). As expected, sHGA, which showed a negative relationship with sNIT, whereas all other serum metabolites showed a positive relationship.

Inevitably there are limitations to this study. Due to the rarity if AKU, we had to rely on analysing all serum and urine samples to generate a large enough number to provide a worthwhile analysis. The time between sampling points was long (usually one year) and daily fluctuations unknown. Derived total body water calculations are based on published methodology but remain pragmatic assumptions. However, for a complex clinical study such as SONIA 2, this methodology is considered practical and acceptable and therefore our data are important and provide valuable insights. Patients were on other medications but a study of this nature cannot exclude the use of concomitant medications where needed; further most medications were taken by patients throughout the duration acting as their own controls^4^. No formal data was collected to exclude heart failure and other conditions which could affect total body water, although adverse events were recorded and did not indicate this factor to be an issue. Hypothyroidism has been shown to cause tyrosinaemia and could be exacerbated by nitisinone^36^, and although all hypothyroid patients were on thyroxine, their thyroid status was not rechecked during SONIA 2. 24-h urine collections are not perfect but are however suited to prospective studies of these kinds, and several recent randomised studies have employed 24-h urine measurements as health outcomes^37,38^. We have also only measured serum concentrations in a single fasting morning sample, since it is impractical to use 24-h blood sampling in such a study design; however, a recent publication has shown that the average 24-h concentrations of metabolites are within 10% of the fasting values and we believe that our analyses provide reliable data^17^.

In summary, analysis of SONIA 2 data in NIT-treated patients reveals a Gaussian distribution of circulating sTYR concentrations in keeping with biological systems. Severe tyrosinaemia is highly prevalent during NIT therapy in SONIA 2. The decrease in conversion of PHE to TYR during NIT-induced tyrosinaemia seems to be an adaptive response to minimise TYR formation. A better understanding of the adaptive response to NIT therapy may possibly translate into a tool to predict keratopathy in the future. The contributions of dietary protein intake and the magnitude of rise in circulating NIT seem to be less influential than decreased conversion of HPPA to HPLA in determining the degree of tyrosinaemia during NIT treatment in SONIA 2.

## Supplementary Information

Supplementary Information.

## Data Availability

SONIA 2 data access will be granted in response to qualified research requests. All de-identified individual participant data, for patients with separate consent signed for this purpose, can be made available to researchers. Data will be shared based on: the scientific merit of the proposal—i.e. the proposal should be scientifically sound, ethical, and have the potential to contribute to the advancement of public health as well as the feasibility of the research proposal—i.e. the requesting research team must be scientifically qualified and have the resources to conduct the proposed project. The data files would exclude data dictionaries that require user licenses. Data could be made available following finalized regulatory authority review and end of any data exclusivity periods and ending after 36 months or until corresponding author is able to fulfil this obligation whichever is earlier. Further, the study protocol and statistical analysis plan can be made available. Proposals should be directed to j.a.gallagher@liverpool.ac.uk to gain access. Data requestors will need to sign a data access agreement.
