# Supplementary material for: Determinants of tyrosinaemia during nitisinone therapy in alkaptonuria
Source: Sci Rep. 2022 Sep 27;12:16083. doi: 10.1038/s41598-022-20424-z (PMC9515198; doi:10.1038/s41598-022-20424-z)
Supplement: Supplementary file 1 — Supplementary Information. [file 41598_2022_20424_MOESM1_ESM.pdf]

**Supplementary material**

**Legend to tables and figures**

**Supplementary tables**

Table S1. Demographic and other data in SONIA 2.

Table S2. Further metabolic data in the control group in SONIA 2 (n = 69)

Table S3. Linear regression relationships of measured data for sTYR, $uUREA_{24}$ and sNIT (linear regression coefficient shown as R and its statistical significance shown by p value).

Table S4. Linear regression relationships of derived data for sTYR, $uUREA_{24}$ and sNIT (linear regression coefficient shown as R and its statistical significance shown by p value).

Table S5. Dietary approach to sTYR in the United Kingdom National Alkaptonuria Centre.

**Supplementary figures**

Figure S1. The tyrosine metabolic pathway is shown highlighting the site of the enzyme defect observed in AKU and the site of action of nitisinone, a reversible competitive inhibitor of 4-hydroxyphenylpyruvate dioxygenase. The pathway also highlights the dynamic relationships between HPPA, TYR and HPLA, a key relationship after nitisinone. (HPPR – 4-hydroxyphenylpyruvate reductase)

Figure S2. Study design of SONIA 2 indicating the visits when assessments including 24-h urine and blood samples were taken.

Figure S3. Changes in sHGA, $uHGA_{24}$, $_{TBW}HGA$, and $cHGA_{24}$ across the sTYR groups of <701, 701-900, 901-1100 and >1100 µmol/L (p values indicated for within-group comparison where statistical significance was achieved).

Figure S4. Changes in sTYR, $uTYR_{24}$, $_{TBW}TYR$, and $cTYR_{24}$ across the sTYR groups of <701, 701-900, 901-1100 and >1100 µmol/L (p values indicated for within-group comparison where statistical significance was achieved).

Figure S5. Changes in sHPPA, $uHPPA_{24}$, $_{TBW}HPPA$, and $cHPPA_{24}$ across the sTYR groups of <701, 701-900, 901-1100 and >1100 µmol/L (p values indicated for within-group comparison where statistical significance was achieved).

Figure S6. Changes in sHPLA, $uHPLA_{24}$, $_{TBW}HPLA$, and $cHPLA_{24}$ across the sTYR groups of <701, 701-900, 901-1100 and >1100 µmol/L (p values indicated for within-group comparison where statistical significance was achieved).

Figure S7. Changes in sHGA/sTYR, $uHGA_{24}/uTYR_{24}$, $_{TBW}HGA/_{TBW}TYR$, and $cHGA_{24}/cTYR_{24}$ across the sTYR groups of <701, 701-900, 901-1100 and >1100 µmol/L (p values indicated for within-group comparison where statistical significance was achieved).

Figure S8. Changes in sTYR/sPHE, $uTYR_{24}/uPHE_{24}$, $_{TBW}TYR/_{TBW}PHE$, and $cTYR_{24}/cPHE_{24}$ across the sTYR groups of <701, 701-900, 901-1100 and >1100 µmol/L (p values indicated for within-group comparison where statistical significance was achieved).

Figure S9. Changes in $uUREA_{24}$, and $uUREA_{24}$/kg across the sTYR groups of <701, 701-900, 901-1100 and >1100 µmol/L (p values indicated for within-group comparison where statistical significance was achieved).

Figure S10. Regression graphs showing relationships between sTYR and $uUREA_{24}$ and sNIT

Figure S11. Regression graphs showing relationships between sTYR and sPHE, sHPPA and sHPLA

## Table S1

| Table S1. Baseline demographic and related variables in the SONIA 2 study [Mean (SD)] | | | | | | |
|---|---|---|---|---|---|---|
| SONIA 2 | | | | | | |
| | Control group | | | Nitisinone group | | |
| | All | Male | Female | All | Male | Female |
| Numbers of patients | 69 | 40 | 29 | 69 | 45 | 24 |
| Age years | 47·7 (10·2) | 48·1 (9·9) | 47 (10·7) | 49 (11·3) | 47·4 (11·9) | 51·9 (9·6) |
| Weight kg | 74·1 (15·6) | 80·4 (13·3) | 65·6 (14·6) | 74·8 (14·8) | 79·2 (12·6) | 66·3 (15·1) |
| Body Mass Index $kg/M^2$ | 26·4 (4·6) | 27 (4·1) | 25·5 (5·2) | 26·9 (4·4) | 27·3 (4·2) | 26·2 (4·7) |
| $uHGA_{24}$ (µmol/day) | 35394 (13868) | 38740 (12282) | 30778 (14797) | 35019 (13124) | 37149 (1258 | 31024 (13447) |
| sHGA (µmol/L) | 28·3 (8·7) | 29·1 (7·7) | 27·1 (9·8) | 30·3 (11) | 31·7 (11·2) | 27·9 (10·4) |
| sTYR (µmol/L) | 64·5 (15·5) | 69·4 (15·3) | 57·8 (13·1) | 65·3 (14·8) | 67 (13·7) | 62·2 (16·6) |

## Table S2

| Table S2. Further metabolic data in the control group in SONIA 2 (n = 69) | | | | | |
|---|---|---|---|---|---|
| | HGA | TYR | PHE | HPPA | HPLA |
| TBW µmol | 1450 (726) | 2942 (1214) | 2701 (887) | <LLoQ | <LLoQ |
| 24-h Urine µmol/day | 32151 (12842) | 165 (134) | 85 (63) | 79 (217) | 72 (98) |
| TBW+URINE µmol | 33590 (13169) | 3113 (1296) | 2778 (910) | 93 (339) | 76 (118) |
| LLoQ – lower limit of quantification | | | | | |

Table S3

| Table S3. Regression analyses of sTYR, $uUREA_{24}$ and sNIT against other measured data | | | | | |
|---|---|---|---|---|---|
| sTYR vs other data | | uUREA24 vs other data | | sNIT vs other data | |
| | R | | R | | R |
| Age years | 0.056 | Age | -0.18** | Age | 0.34**** |
| Weight kg | -0.085 | Weight kg | 0.27**** | Weight kg | -0.42**** |
| $uUREA_{24}$ mmol/day | 0.12* | $uUREA_{24}$ mmol/day | | $uUREA_{24}$ mmol/day | 0.29**** |
| uUREA mmol/kg | 0.12* | uUREA mmol/kg | | uUREA mmol/kg | |
| | | | | | |
| sHGA umol/L | 0.024 | sHGA umol/L | 0.15* | sHGA umol/L | -0.37**** |
| sTYR umol/L | | sTYR umol/L | | sTYR umol/L | 0.21*** |
| sPHE umol/L | 0.31**** | sPHE umol/L | 0.16* | sPHE umol/L | 0.13* |
| sHPPA umol/L | 0.28**** | sHPPA umol/L | 0.003 | sHPPA umol/L | 0.18** |
| sHPLA umol/L | 0.59**** | sHPLA umol/L | 0.07 | sHPLA umol/L | 0.51**** |
| sNIT umol/L | 0.21**** | sNIT umol/L | -0.29**** | sNIT umol/L | |
| | | | | | |
| $uHGA_{24}$ umol/day | -0.06 | $uHGA_{24}$ umol/day | 0.21*** | $uHGA_{24}$ umol/day | -0.4**** |
| $uTYR_{24}$ umol/day | 0.09 | $uTYR_{24}$ umol/day | 0.59**** | $uTYR_{24}$ umol/day | -0.23**** |
| $uPHE_{24}$ umol/day | -0.05 | $uPHE_{24}$ umol/day | 0.54**** | $uPHE_{24}$ umol/day | -0.16** |
| $uHPPA_{24}$ umol/day | 0.1 | $uHPPA_{24}$ umol/day | 0.8**** | $uHPPA_{24}$ umol/day | -0.18** |
| $uHPLA_{24}$ umol/day | 0.23**** | $uHPLA_{24}$ umol/day | 0.75**** | $uHPLA_{24}$ umol/day | -0.07 |
| Degree of statistical significance of R is indicated by p<: *0.05; **<0.01; ***<0.001; ****<0.0001 | | | | | |

# Table S4

| Table S4. Regression analyses of sTYR, $uUREA_{24}$ and sNIT against other derived data | | | | | |
|---|---|---|---|---|---|
| sTYR vs other data | | uUREA24 vs other data | | sNIT vs other data | |
| | R | | R | | R |
| $_{TBW}$HGA umol | 0.02 | $_{TBW}$HGA umol | 0.16** | $_{TBW}$HGA umol | -0.39**** |
| $_{TBW}$TYR umol | 0.71**** | $_{TBW}$TYR umol | 0.28**** | $_{TBW}$TYR umol | -0.13* |
| $_{TBW}$PHE umol | 0.14* | $_{TBW}$HE umol | 0.26**** | $_{TBW}$PHE umol | -0.16** |
| $_{TBW}$HPPA umol | 0.22**** | $_{TBW}$HPPA umol | 0.14* | $_{TBW}$HPPA umol | -0.01 |
| $_{TBW}$HPLA umol | 0.47**** | $_{TBW}$HPLA umol | 0.23**** | $_{TBW}$HPLA umol | 0.2*** |
| | | | | | |
| $cHGA_{24}$ umol/day | -0.05 | $cHGA_{24}$ umol/day | 0.21*** | $cHGA_{24}$ umol/day | -0.42**** |
| $cTYR_{24}$ umol/day | 0.2**** | $cTYR_{24}$ umol/day | 0.31**** | $cTYR_{24}$ umol/day | -0.14* |
| $cPHE_{24}$ umol/day | 0.16** | $cPHE_{24}$ umol/day | 0.28**** | $cPHE_{24}$ umol/day | -0.18** |
| $cHPPA_{24}$ umol/day | 0.12* | $cHPPA_{24}$ umol/day | 0.79**** | $cHPPA_{24}$ umol/day | -0.18** |
| $cHPLA_{24}$ umol/day | 0.31**** | $cHPLA_{24}$ umol/day | 0.72**** | $cHPLA_{24}$ umol/day | -0.01 |
| Degree of statistical significance of R is indicated by p<: *0.05; **<0.01; ***<0.001; ****<0.0001 | | | | | |

Table S5

| Table S5. Serum tyrosine thresholds used for dietetic intervention in the NAC during nitisinone | |
|---|---|
| sTYR (µmol/L) | Action |
| <500 | Acceptable, no further action |
| 501 – 700 | Institute 0·9g/kg body weight protein in diet |
| 701 – 900 | Institute 0·8g/kg body weight protein in diet |
| >900 | Institute 0·8g/kg body weight protein in diet, plus tyrosine/phenylalanine-free amino-acid supplements |
| Keratopathy | Stop nitisinone and restart after 2months, intensify dietary protein restriction |

Figure S1

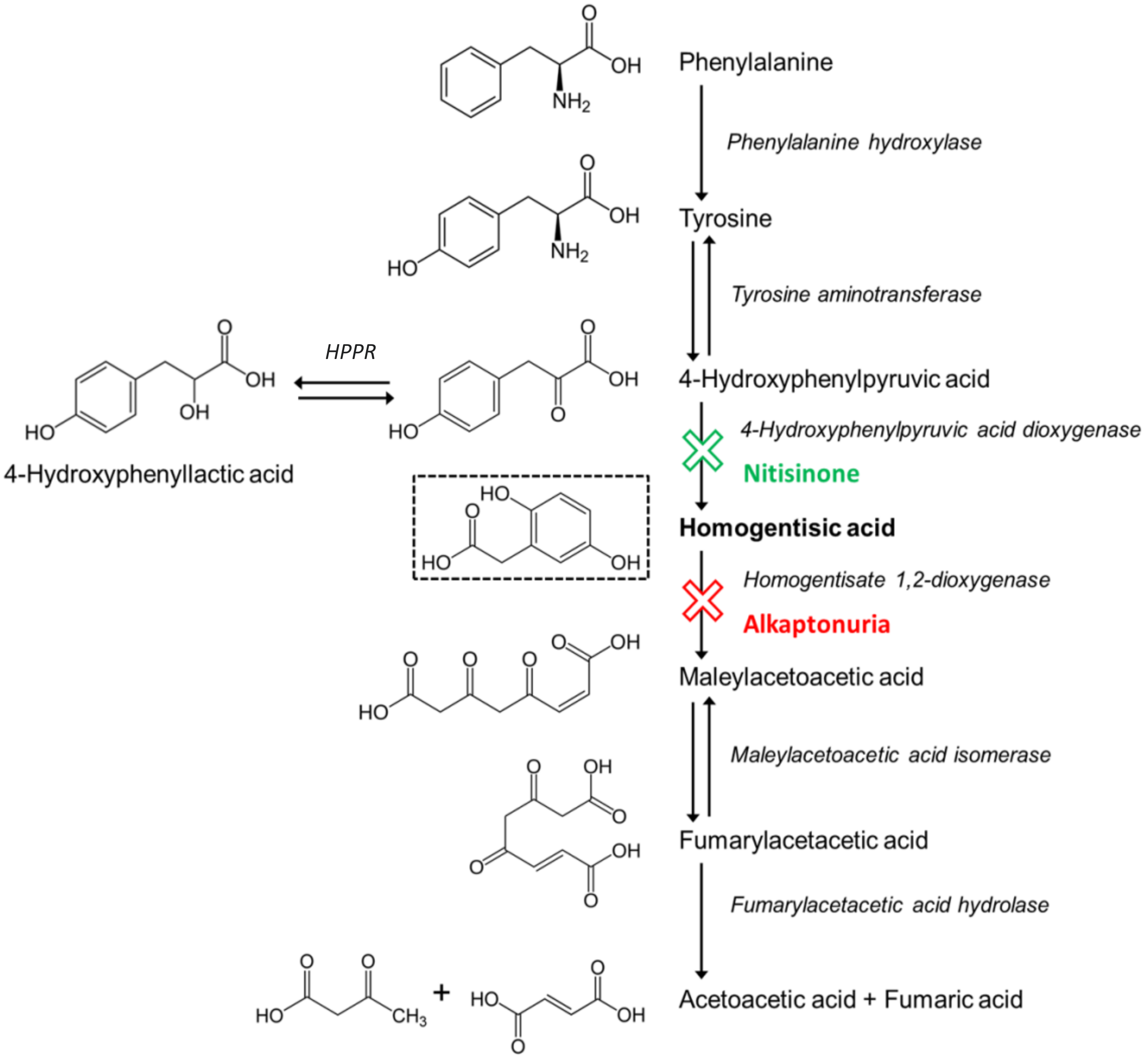

Phenylalanine
Phenylalanine hydroxylase
Tyrosine
Tyrosine aminotransferase
HPPR
4-Hydroxyphenyllactic acid
4-Hydroxyphenylpyruvic acid
4-Hydroxyphenylpyruvic acid dioxygenase
Nitisinone
Homogentisic acid
Homogentisate 1,2-dioxygenase
Alkaptonuria
Maleylacetoacetic acid
Maleylacetoacetic acid isomerase
Fumarylacetacetic acid
Fumarylacetacetic acid hydrolase
Acetoacetic acid + Fumaric acid

Figure S2

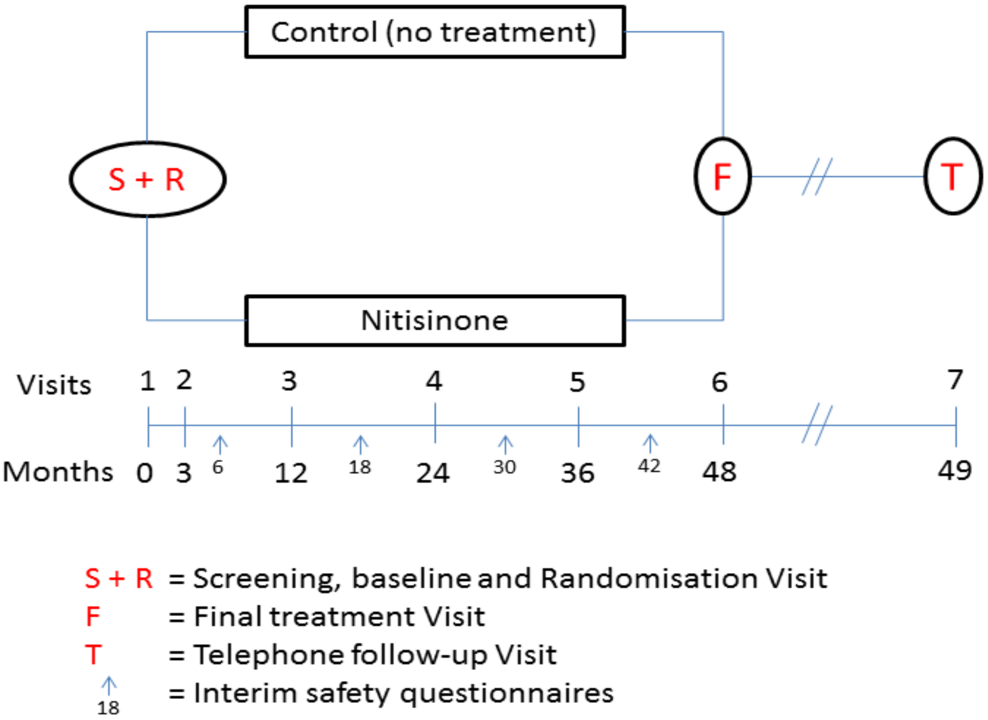
Control (no treatment)
S + R
F
T
Nitisinone
Visits 1 2 3 4 5 6 7
Months 0 3 6 12 18 24 30 36 42 48 49
S + R = Screening, baseline and Randomisation Visit
F = Final treatment Visit
T = Telephone follow-up Visit
18 = Interim safety questionnaires

Figure S3

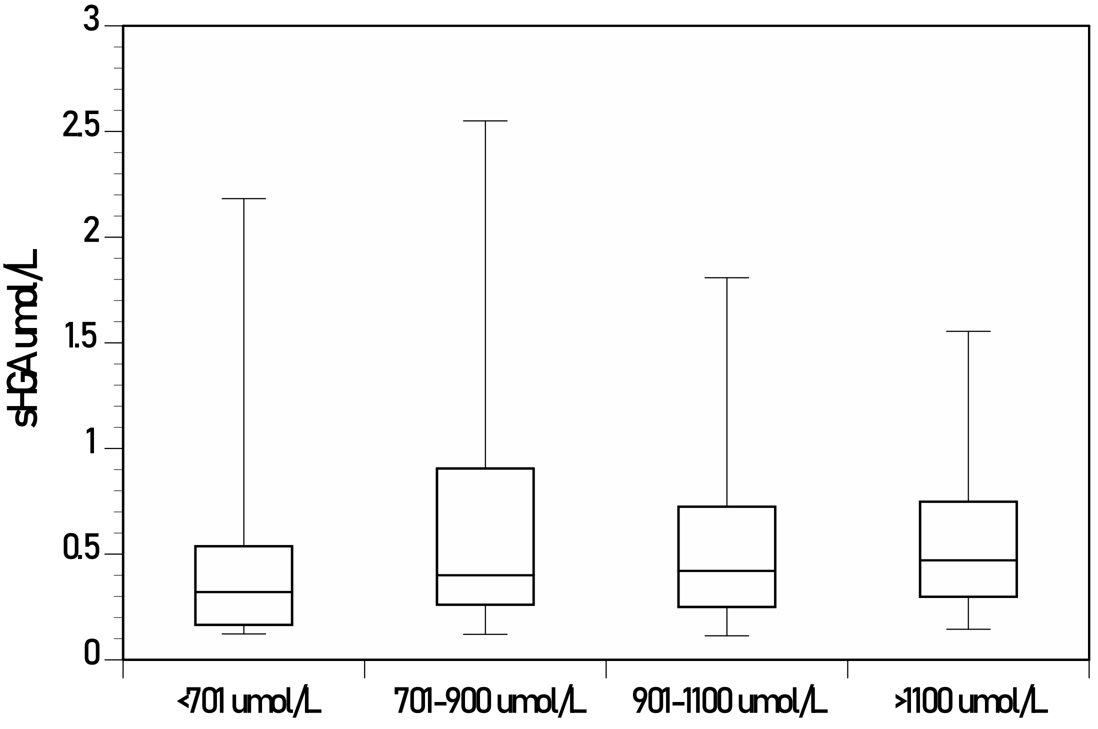

3
2.5
2
1.5
1
0.5
0
sHGA umol/L
<701 umol/L
701-900 umol/L
901-1100 umol/L
>1100 umol/L


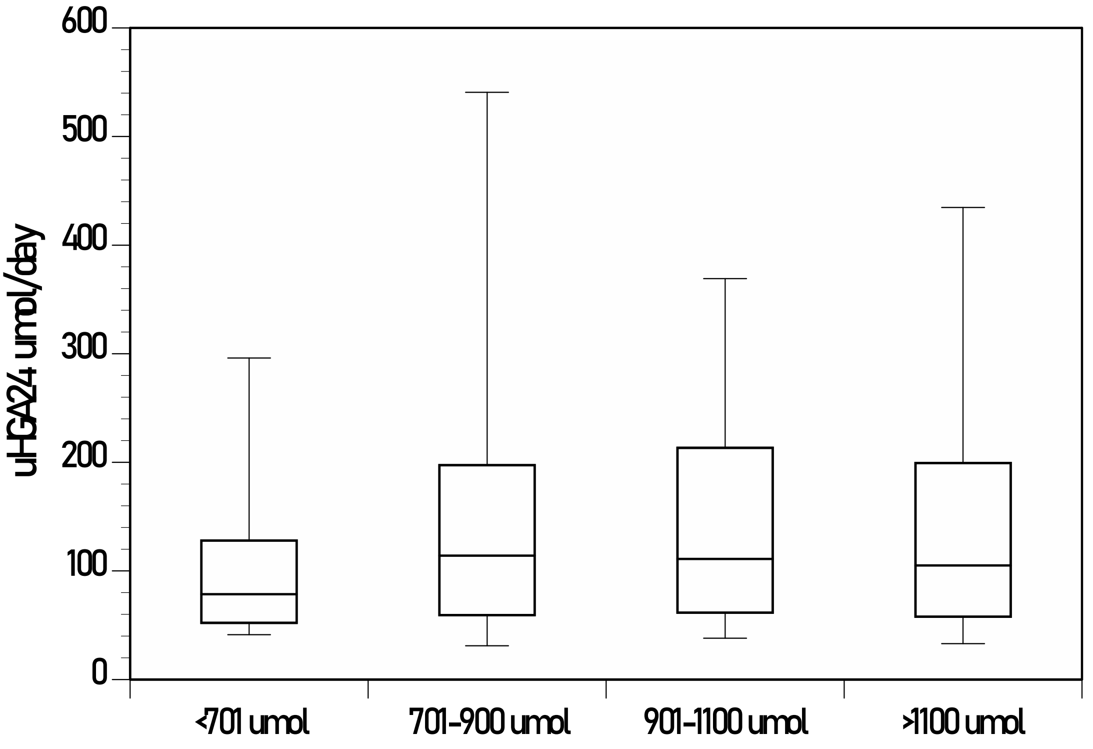

600
500
400
300
200
100
0
uHGA24 umol/day
<701 umol
701-900 umol
901-1100 umol
>1100 umol

Figure S3

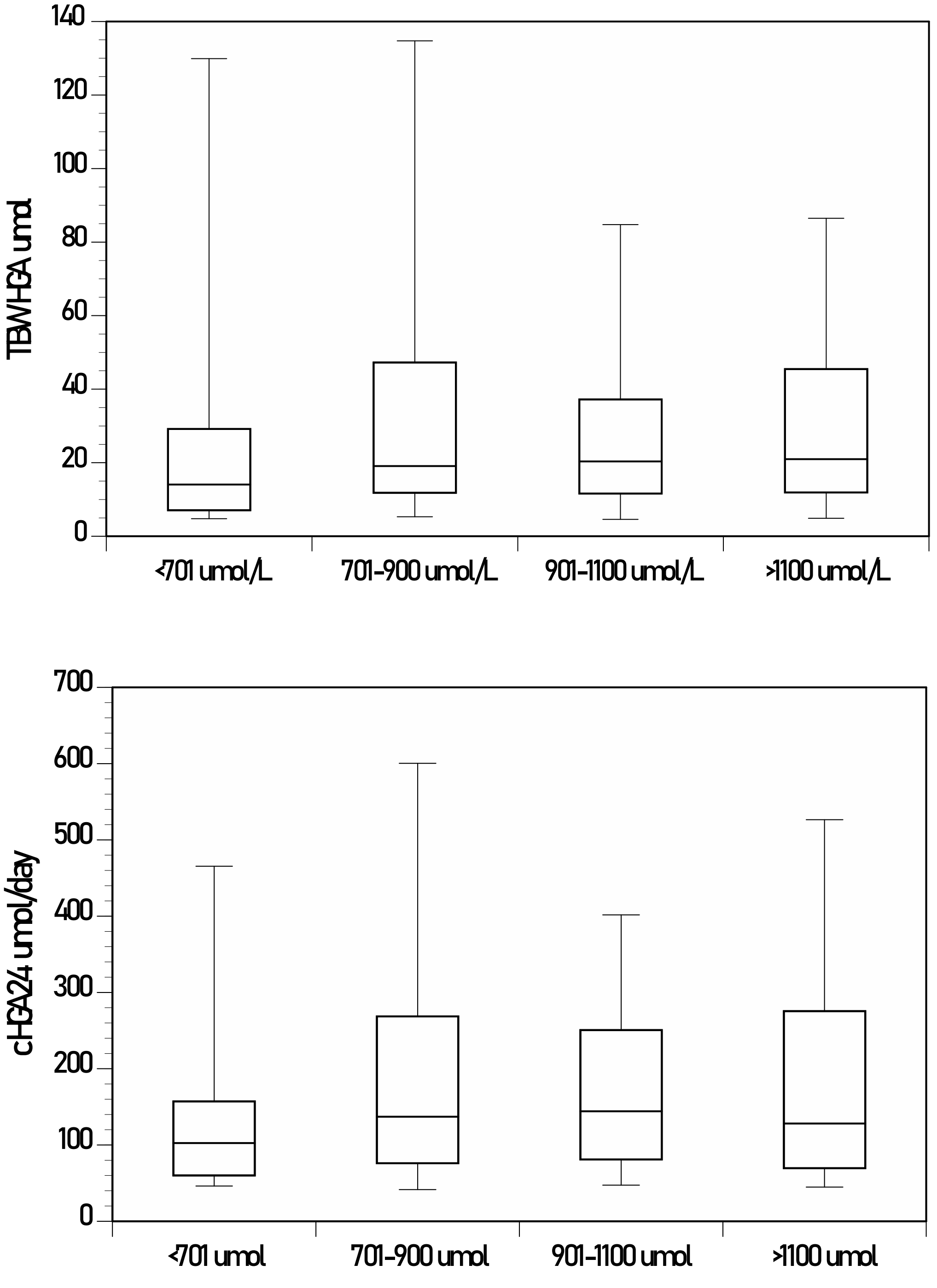
TBWHGA umol
140
120
100
80
60
40
20
0
<701 umol/L
701-900 umol/L
901-1100 umol/L
>1100 umol/L
cHGA24 umol/day
700
600
500
400
300
200
100
0
<701 umol
701-900 umol
901-1100 umol
>1100 umol

Figure S4

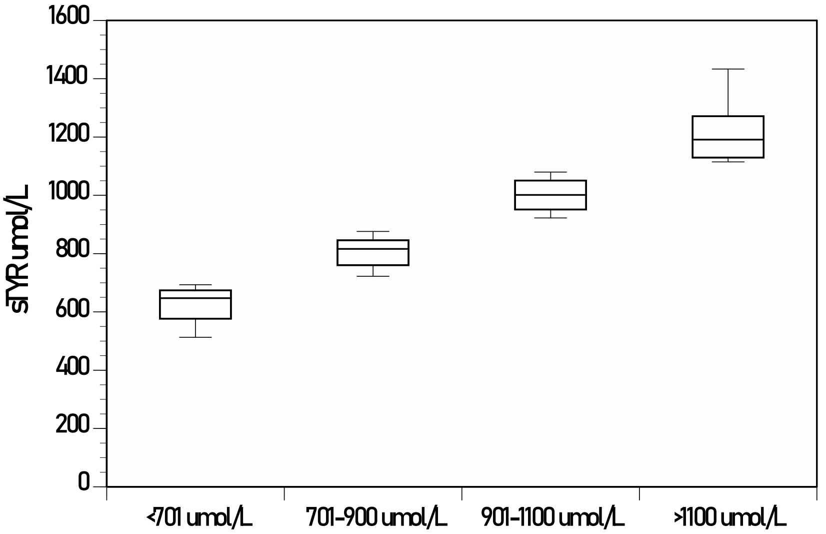


<701 vs 701-900 p<0.0001     <701 vs 901-1100 p<0.0001

<701 vs >1100 p<0.0001     701-900 vs 901-1100 p<0.0001

701-900 vs >1100 p<0.0001     901-1100 vs >1100 p<0.0001

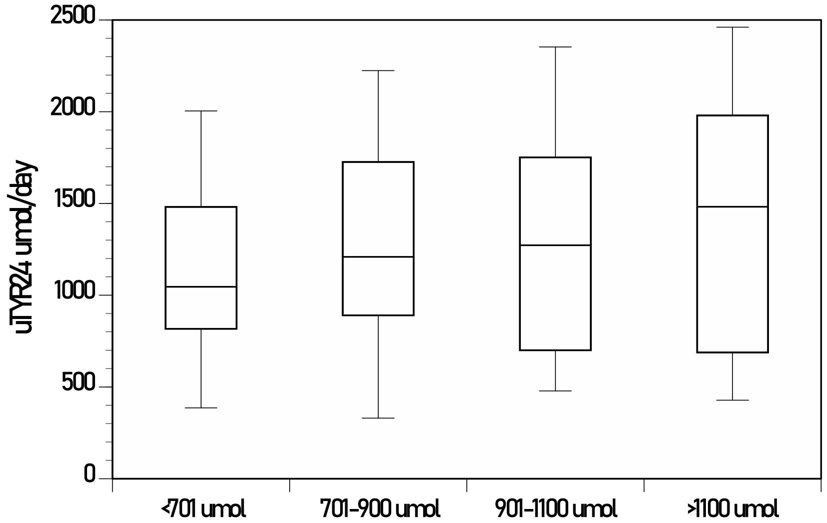

Figure S4

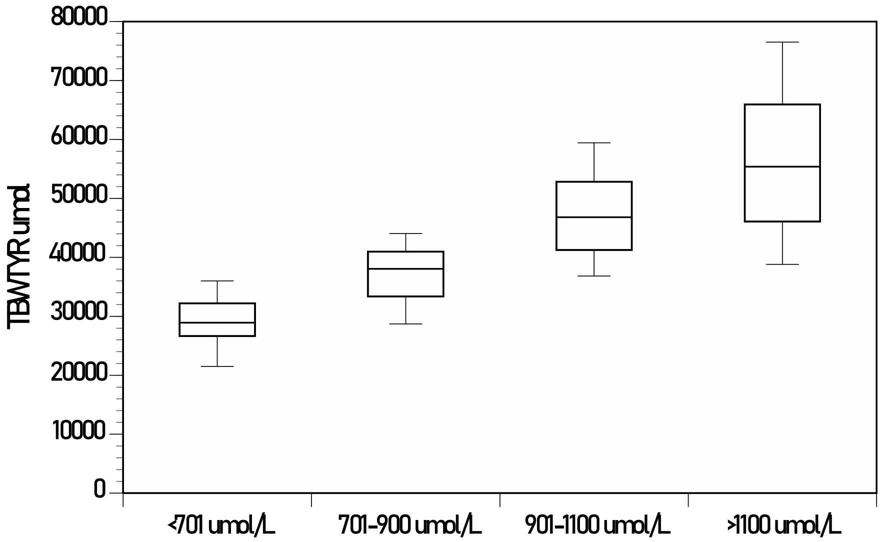


<701 vs 701-900 p<0.0001

<701 vs 901-1100 p<0.0001

<701 vs >1100 p<0.0001

701-900 vs 901-1100 p<0.0001

701-900 vs >1100 p<0.0001

901-1100 vs >1100 p<0.0001

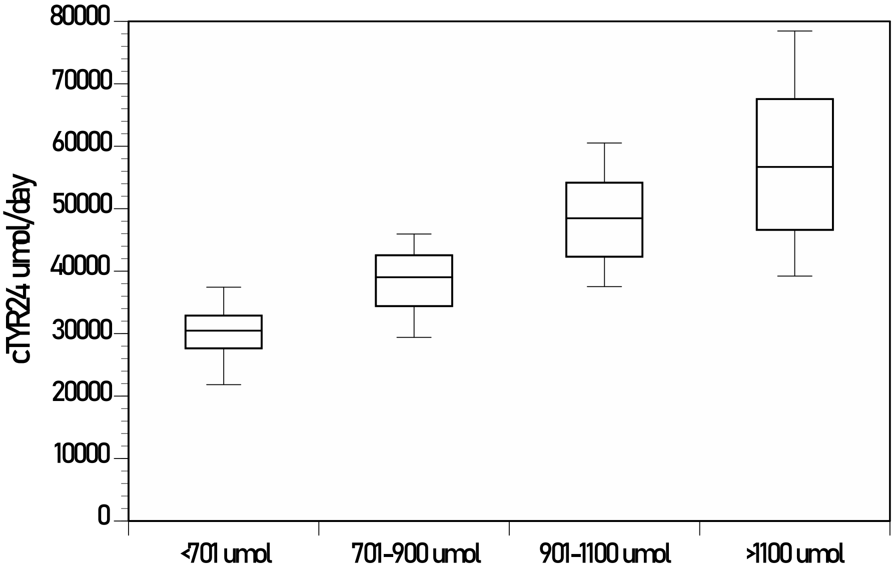


<701 vs 701-800 p<0.0001

<701 vs 901-1100 p<0.0001

<701 vs >1100 p<0.0001

701-900 vs 901-1100 p<0.0001

701-900 vs >1100 p<0.0001

901-1100 vs >1100 p<0.0001

Figure S5

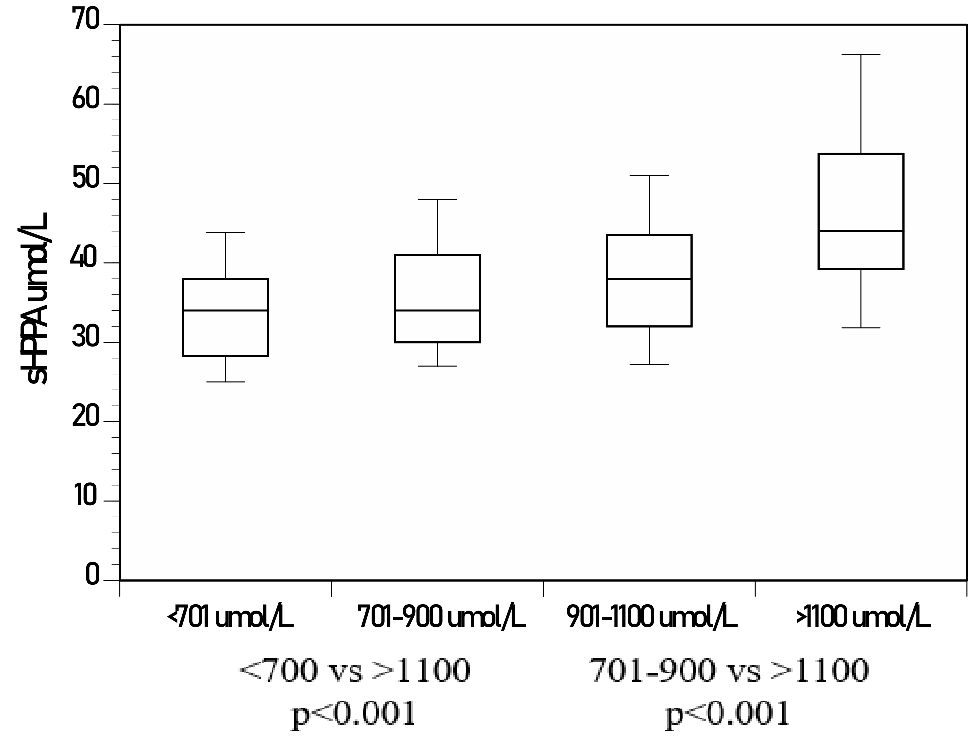
sHPPA umol/L
0
10
20
30
40
50
60
70
<701 umol/L
701-900 umol/L
901-1100 umol/L
>1100 umol/L
<700 vs >1100
p<0.001
701-900 vs >1100
p<0.001

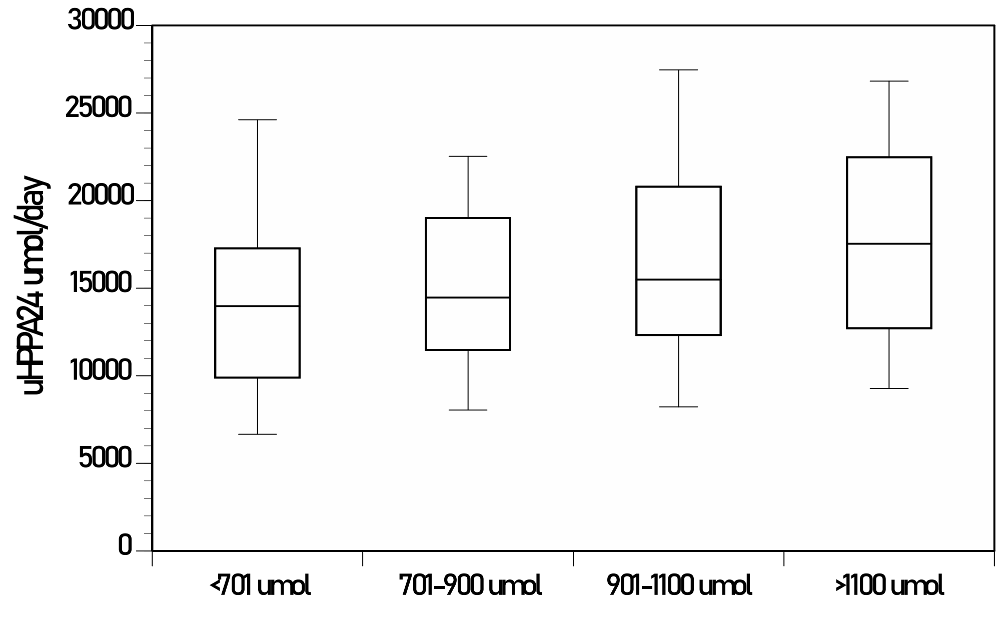
uHPPA24 umol/day
0
5000
10000
15000
20000
25000
30000
<701 umol
701-900 umol
901-1100 umol
>1100 umol

Figure S5

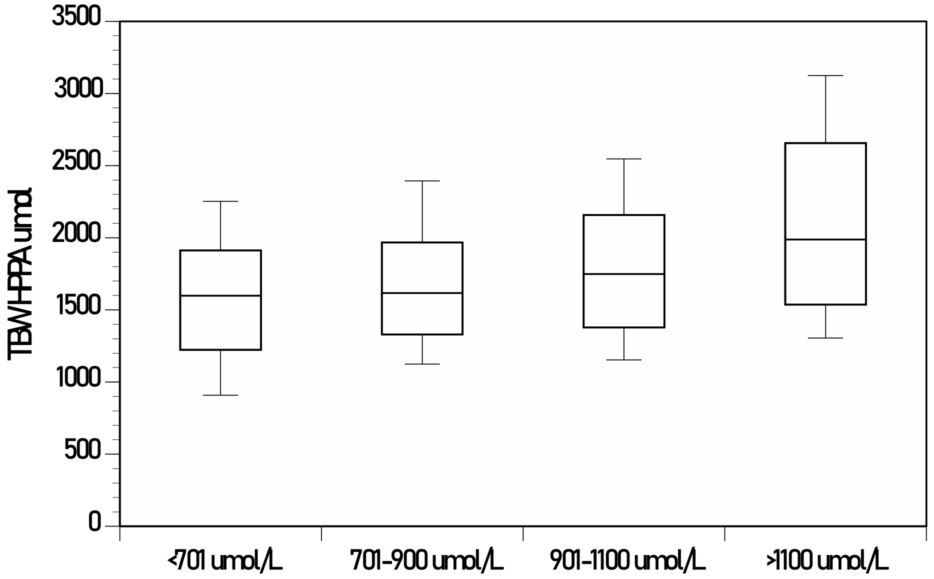


<700 vs >1100 $p<0.001$

701-900 vs >1100 $p<0.001$

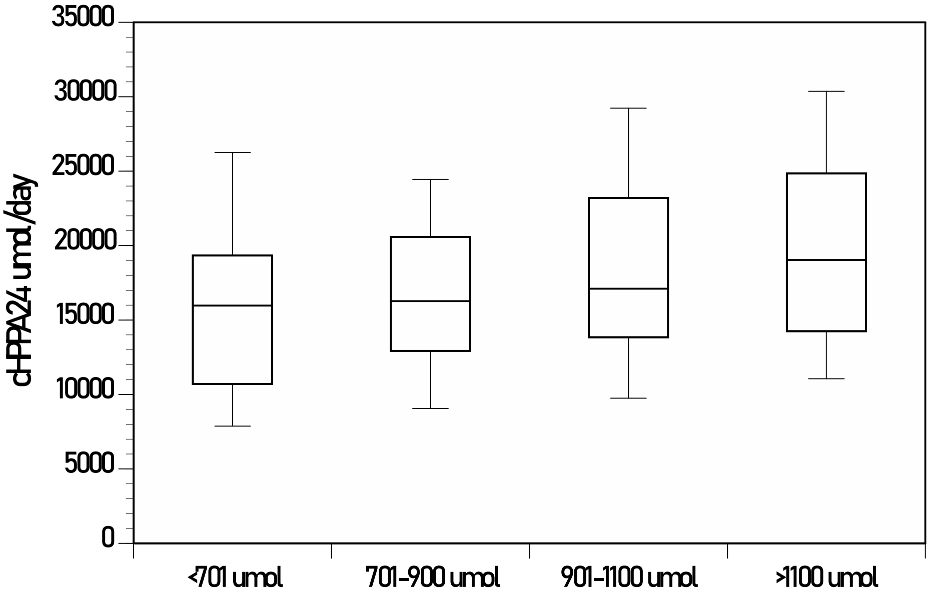

Figure S6

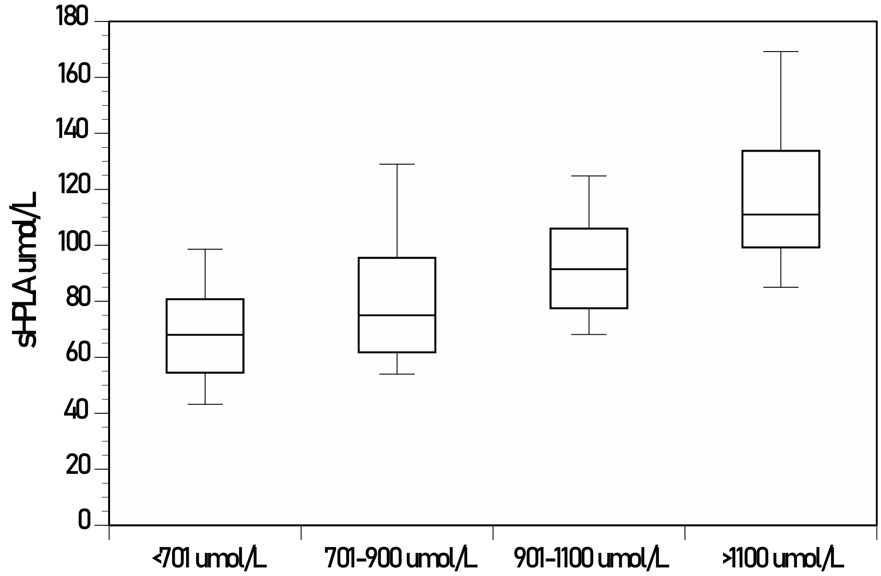


<701 vs 701-900 p<0.05     <701 vs 901-1100 p<0.001

<701 vs >1100 p<0.001     701-900 vs 901-1100 p<0.05

701-900 vs >1100 p<0.001     901-1100 vs >1100 p<0.001

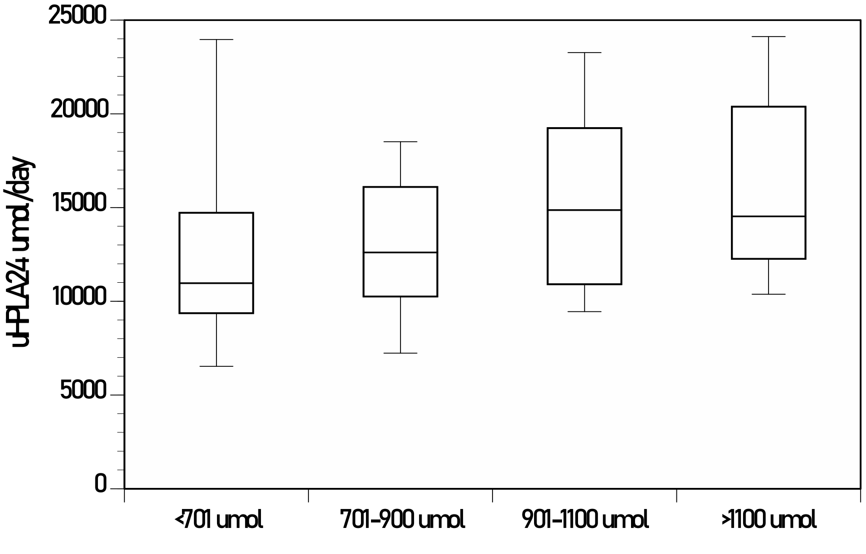


<701 vs 901-1100 p<0.05     <701 vs >1100 p<0.01

701-900 vs 901-1100 p<0.05     701-900 vs >1100 p<0.01

Figure S6

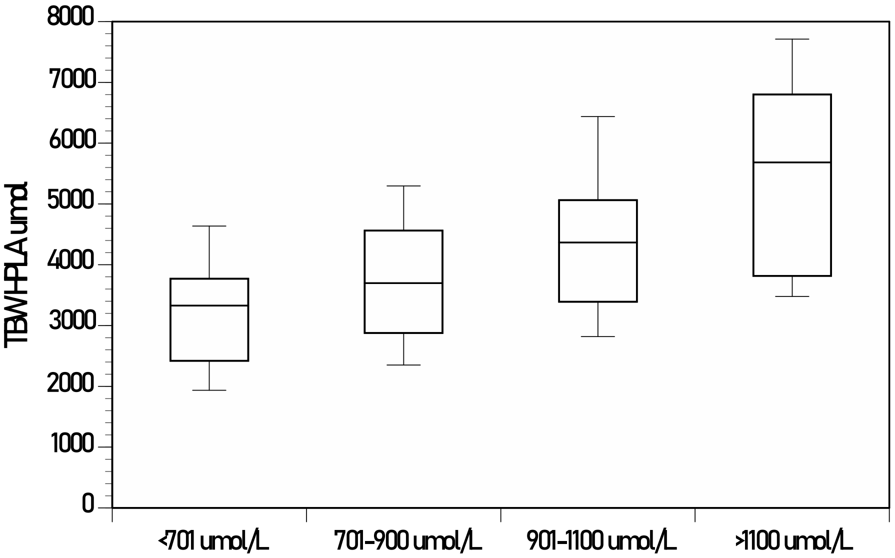


<701 vs 901-1100 p<0.001

<701 vs >1100 p<0.001

701-900 vs 901-1100 p<0.05

701-900 vs >1100 p<0.001

901-1100 vs >1100 p<0.001

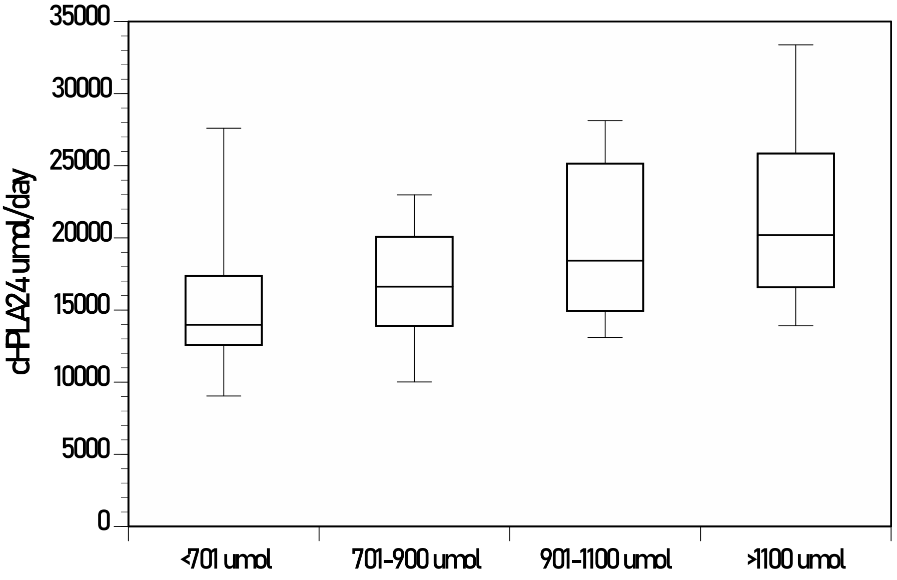


<701 vs 901-1100 p<0.001

<701 vs >1100 p<0.001

701-900 vs 901-1100 p<0.05

701-900 vs >1100 p<0.001

Figure S7

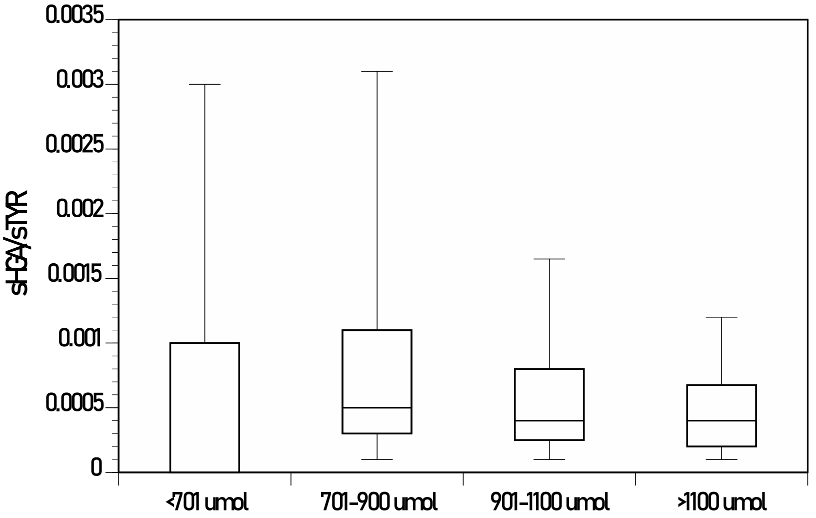

sHGA/sTYR
0.0035
0.003
0.0025
0.002
0.0015
0.001
0.0005
0
<701 umol
701-900 umol
901-1100 umol
>1100 umol


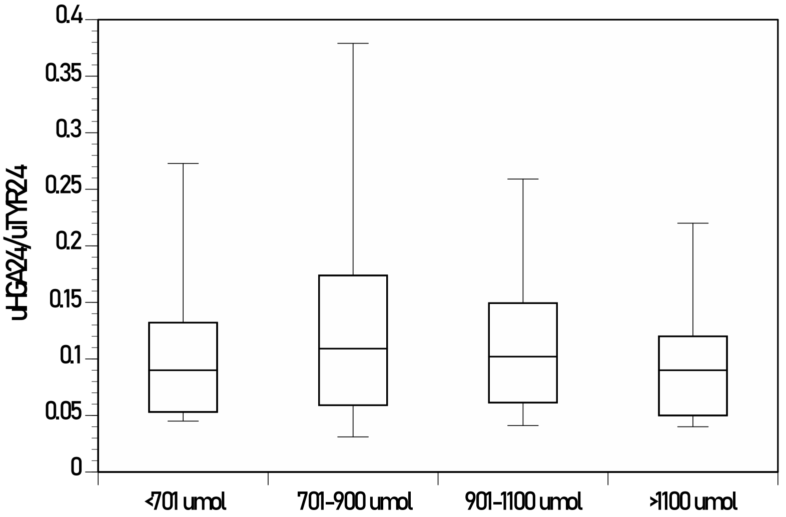

uHGA24/uTYR24
0.4
0.35
0.3
0.25
0.2
0.15
0.1
0.05
0
<701 umol
701-900 umol
901-1100 umol
>1100 umol

Figure S7

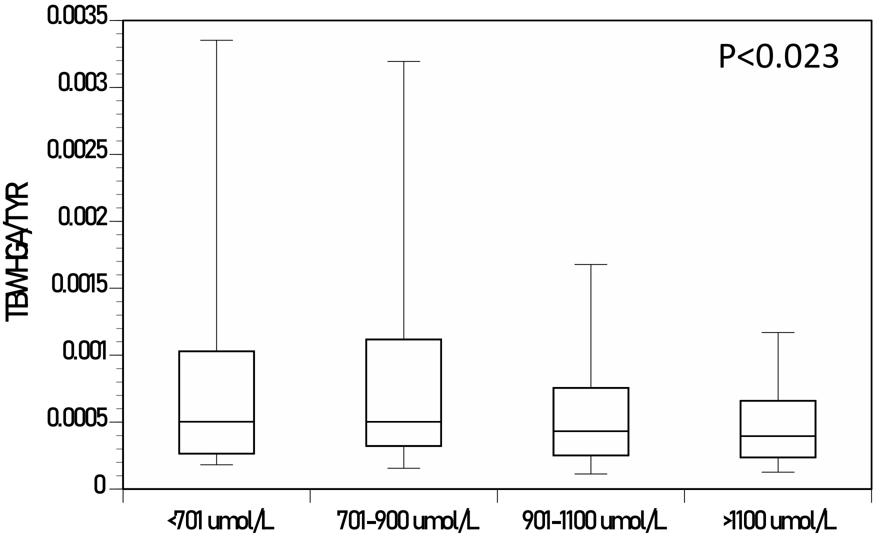

P<0.023
TBWHGA/TYR
0.0035
0.003
0.0025
0.002
0.0015
0.001
0.0005
0
<701 umol/L
701-900 umol/L
901-1100 umol/L
>1100 umol/L


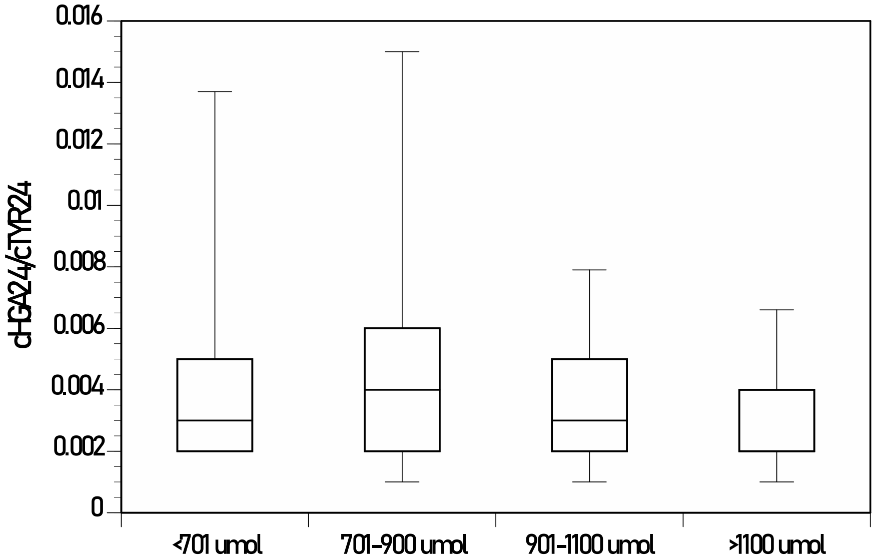

cHGA24/cTYR24
0.016
0.014
0.012
0.01
0.008
0.006
0.004
0.002
0
<701 umol
701-900 umol
901-1100 umol
>1100 umol

Figure S8

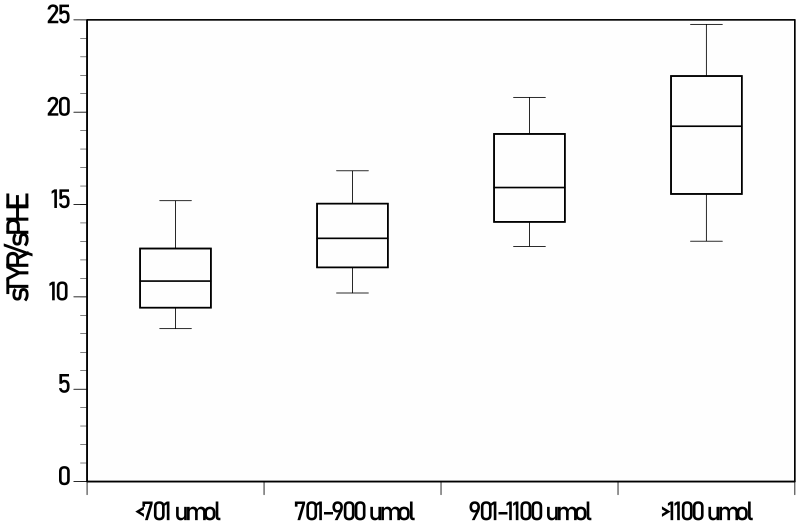


<701 vs 701-800 p<0.0001 <701 vs 901-1100 p<0.0001

<701 vs >1100 p<0.0001 701-900 vs 901-1100 p<0.0001

701-900 vs >1100 p<0.0001 901-1100 vs >1100 p<0.0001

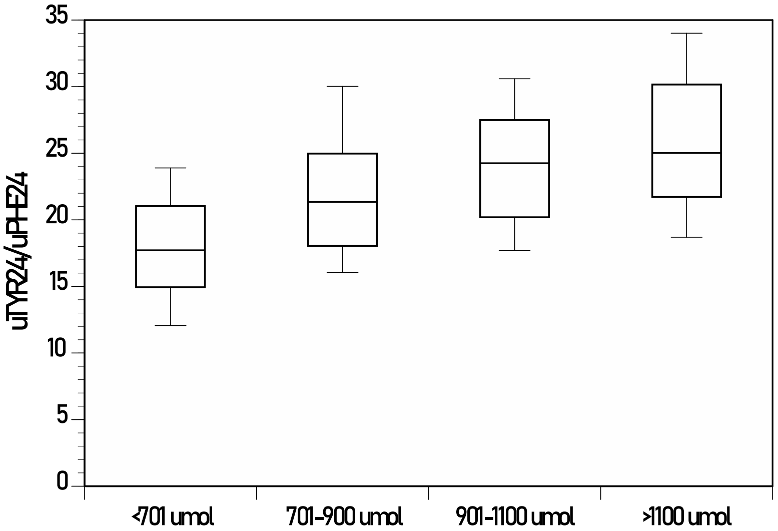


<701 vs 701-900 p<0.001 <701vs 901-1100 p<0.001

<701 vs >1100 p<0.001 701-900 vs 901-1100 p<0.05

701-900 vs >1100 p<0.001

Figure S8

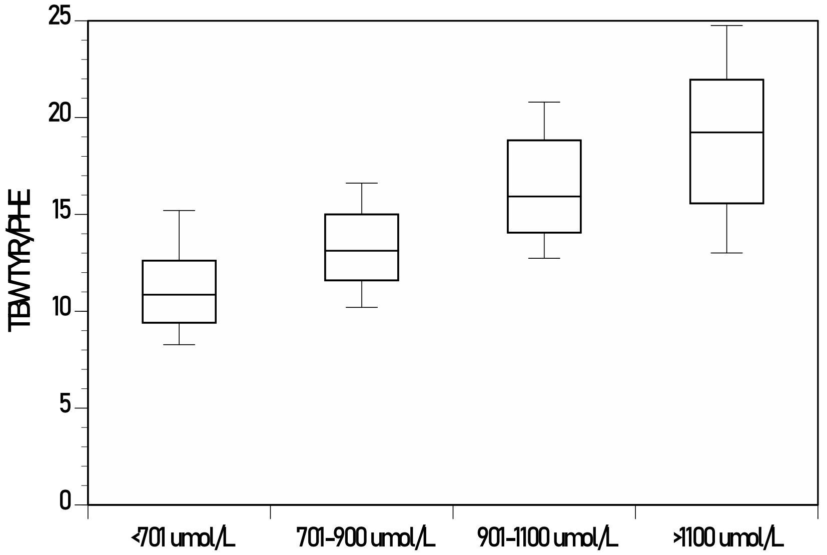


<701 vs 701-800 p<0.0001

<701 vs 901-1100 p<0.0001

<701 vs >1100 p<0.0001

701-900 vs 901-1100 p<0.0001

701-900 vs >1100 p<0.0001

901-1100 vs >1100 p<0.0001

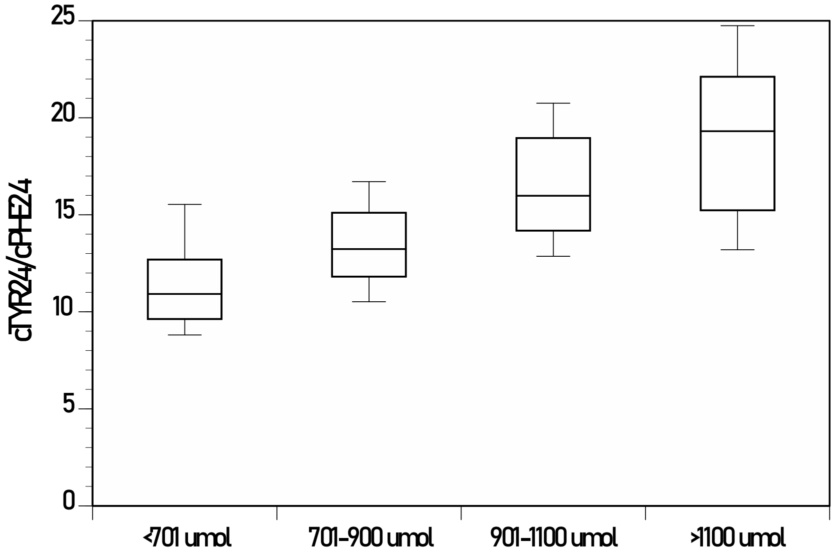


<701 vs 701-800 p<0.0001

<701 vs 901-1100 p<0.0001

<701 vs >1100 p<0.0001

701-900 vs 901-1100 p<0.0001

701-900 vs >1100 p<0.0001

901-1100 vs >1100 p<0.0001

Figure S9

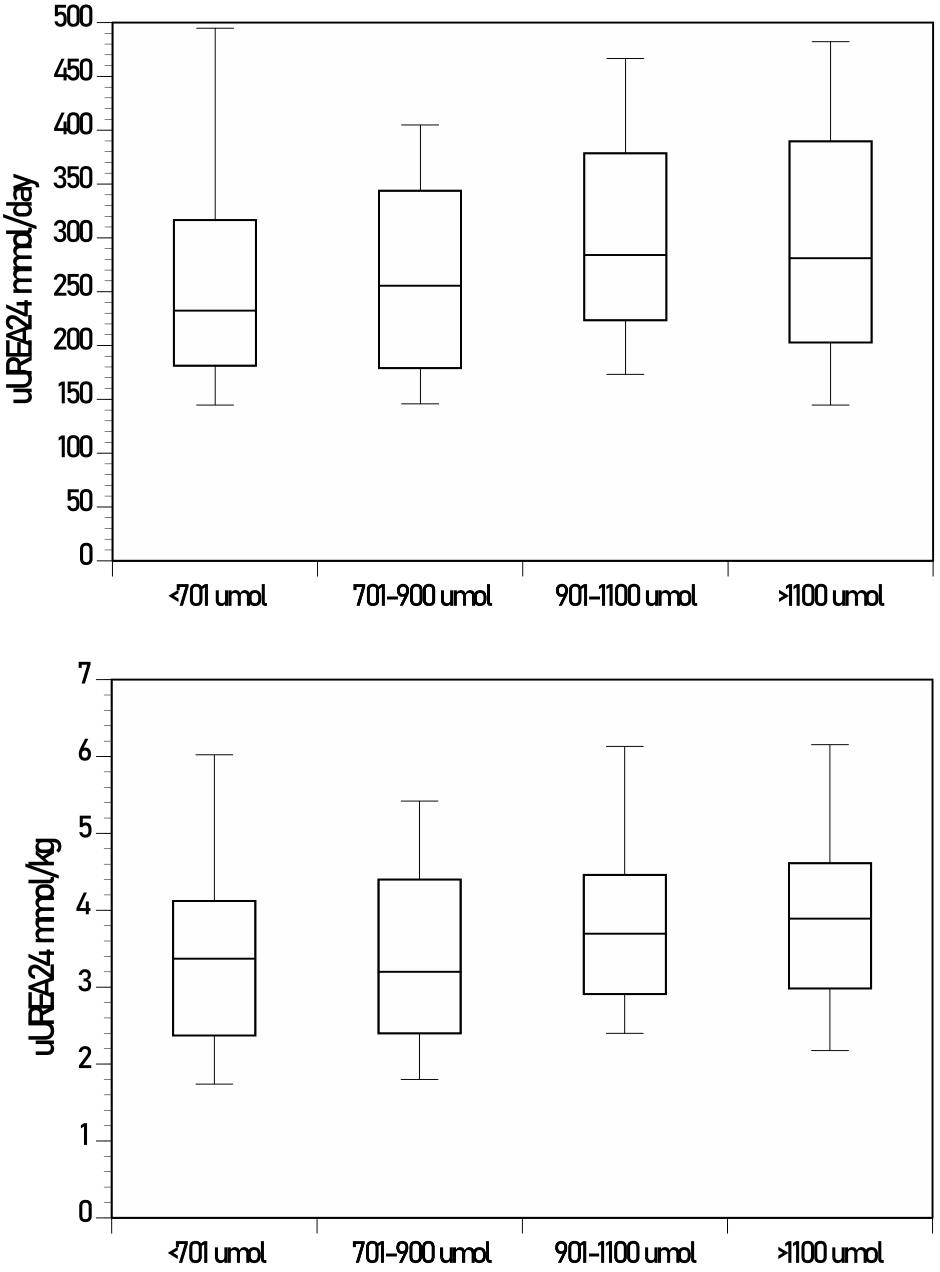
uUREA24 mmol/day
500
450
400
350
300
250
200
150
100
50
0
<701 umol
701-900 umol
901-1100 umol
>1100 umol
uUREA24 mmol/kg
7
6
5
4
3
2
1
0
<701 umol
701-900 umol
901-1100 umol
>1100 umol

Figure S10

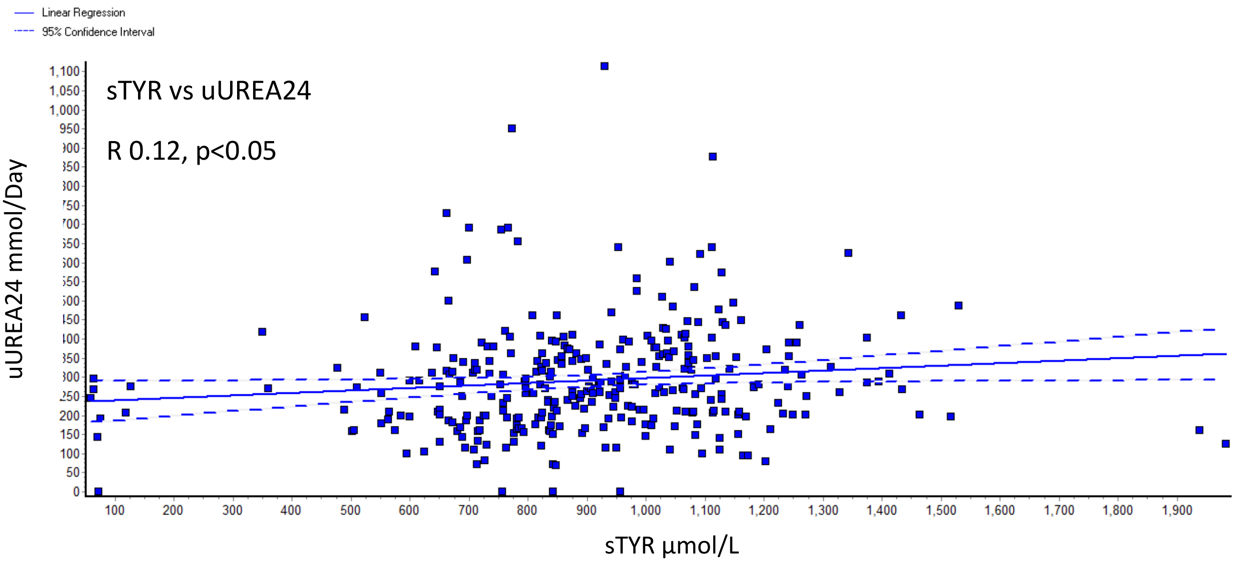

Linear Regression
95% Confidence Interval
sTYR vs uUREA24
R 0.12, p<0.05
uUREA24 mmol/Day
sTYR µmol/L


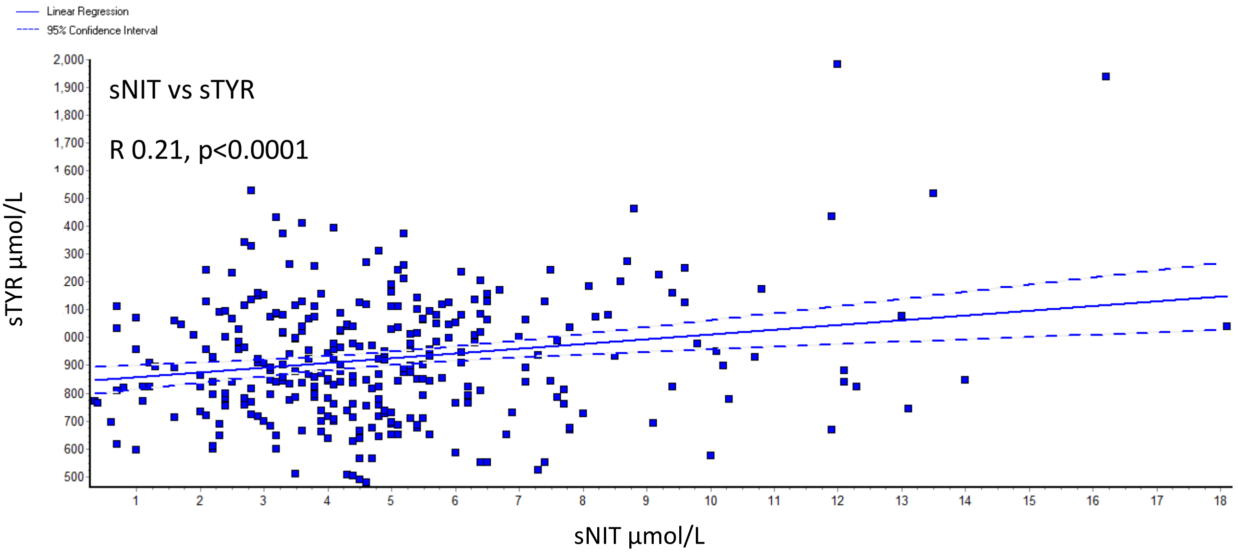

Linear Regression
95% Confidence Interval
sNIT vs sTYR
R 0.21, p<0.0001
sTYR µmol/L
sNIT µmol/L

Figure S11

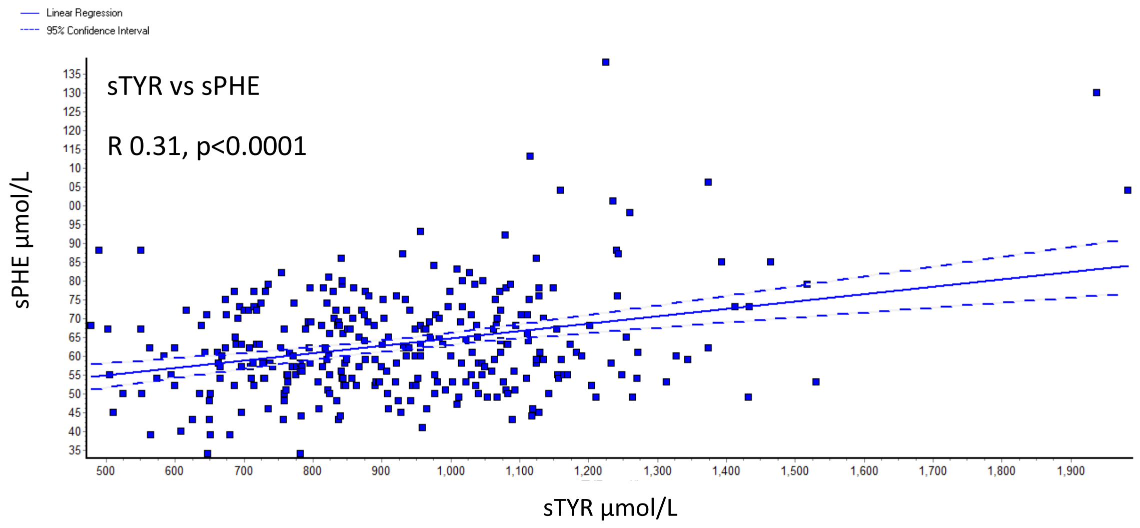
Linear Regression
95% Confidence Interval
sTYR vs sPHE
R 0.31, p<0.0001
sPHE µmol/L
sTYR µmol/L

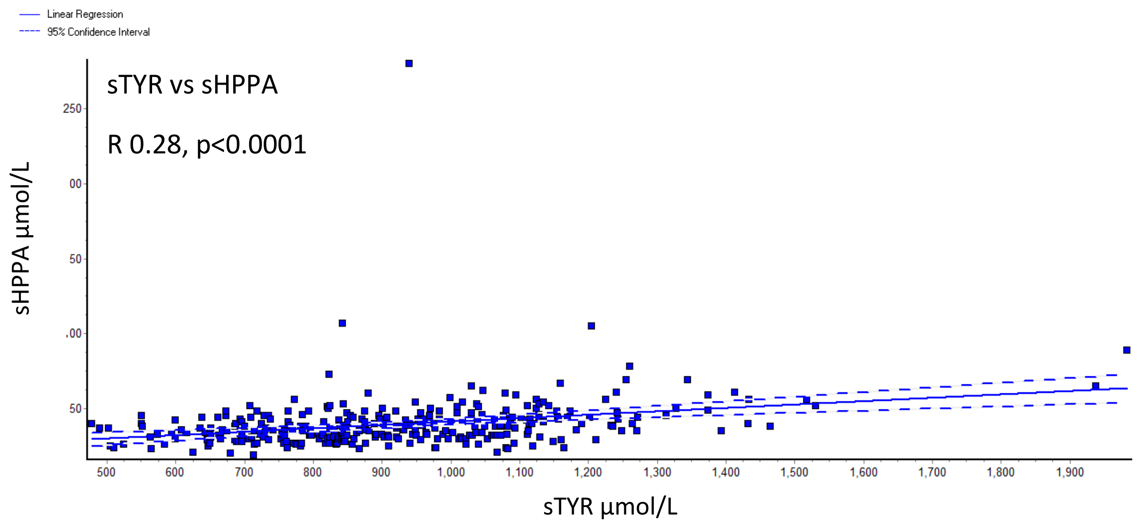
Linear Regression
95% Confidence Interval
sTYR vs sHPPA
R 0.28, p<0.0001
sHPPA µmol/L
sTYR µmol/L

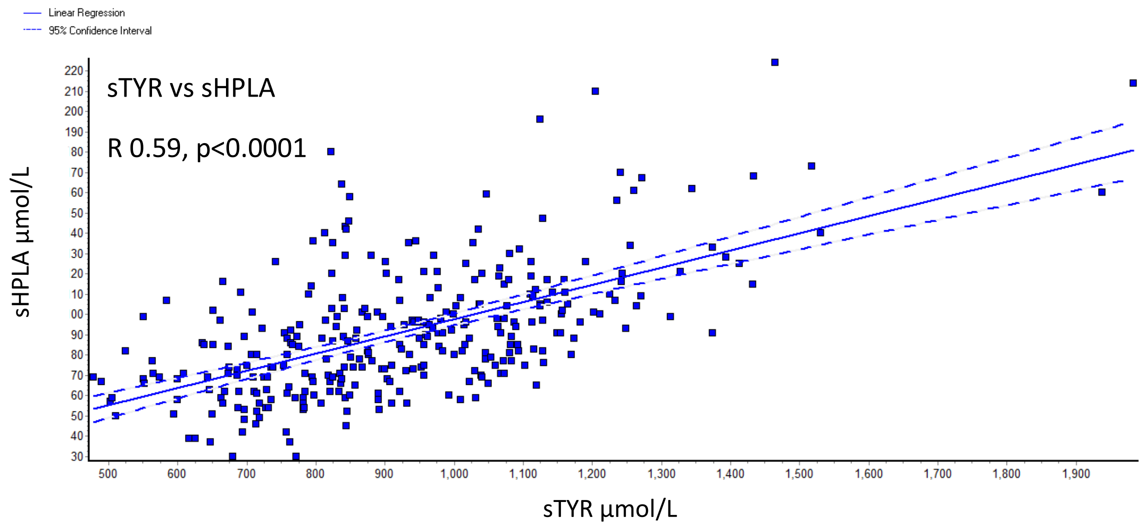
Linear Regression
95% Confidence Interval
sTYR vs sHPLA
R 0.59, p<0.0001
sHPLA µmol/L
sTYR µmol/L
